# Effects of large‐scale disturbance on animal space use: Functional responses by greater sage‐grouse after megafire

**DOI:** 10.1002/ece3.9933

**Published:** 2023-04-07

**Authors:** Bryan S. Stevens, Shane B. Roberts, Courtney J. Conway, Devin K. Englestead

**Affiliations:** ^1^ Idaho Cooperative Fish and Wildlife Research Unit, Department of Fish and Wildlife Sciences University of Idaho Moscow Idaho USA; ^2^ Idaho Department of Fish and Game Boise Idaho USA; ^3^ U.S. Geological Survey, Idaho Cooperative Fish and Wildlife Research Unit University of Idaho Moscow Idaho USA; ^4^ U.S. Bureau of Land Management Idaho Falls Idaho USA

**Keywords:** *Centrocercus urophasianus*, habitat model, habitat selection, habitat use, predicting habitat quality, resource selection function

## Abstract

Global change has altered the nature of disturbance regimes, and megafire events are increasingly common. Megafires result in immediate changes to habitat available to terrestrial wildlife over broad landscapes, yet we know surprisingly little about how such changes shape space use of sensitive species in habitat that remains. Functional responses provide a framework for understanding and predicting changes in space use following habitat alteration, but no previous studies have assessed functional responses as a consequence of megafire. We studied space use and tested for functional responses in habitat use by breeding greater sage‐grouse (*Centrocercus urophasianus*) before and after landscape‐level changes induced by a >40,000 ha, high‐intensity megafire that burned sagebrush steppe in eastern Idaho, USA. We also incorporated functional responses into predictive resource selection functions (RSFs) to map breeding habitat before and after the fire. Megafire had strong effects on the distribution of available resources and resulted in context‐dependent habitat use that was heterogeneous across different components of habitat. We observed functional responses in the use and selection of a variety of resources (shrubs and herbaceous vegetation) for both nesting and brood rearing. Functional responses in the use of nesting habitat were influenced by the overarching effect of megafire on vegetation, whereas responses during brood rearing appeared to be driven by individual variation in available resources that were conditional on nest locations. Importantly, RSFs built using data collected prior to the burn also had poor transferability for predicting space use in a post‐megafire landscape. These results have strong implications for understanding and predicting how animals respond to a rapidly changing environment, given that increased severity, frequency, and extent of wildfire are consequences of global change with the capacity to reshape ecosystems. We therefore demonstrate a conceptual framework to better understand space use and aid habitat conservation for wildlife in a rapidly changing world.

## Graphical abstract

We studied space use of breeding greater sage‐grouse before and after landscape‐level changes to nesting and brood‐rearing habitats that were induced by a megafire event that burned sagebrush steppe in eastern Idaho, USA. Megafire had strong effects on the distribution of resources and resulted in context‐dependent habitat use and selection that were heterogeneous across different components of sage‐grouse habitat. Functional responses in the use and selection of nesting habitat were shaped by the overarching effect of megafire on vegetation, and models built using data collected prior to the megafire had poor transferability for predicting space use in a post‐megafire landscape.

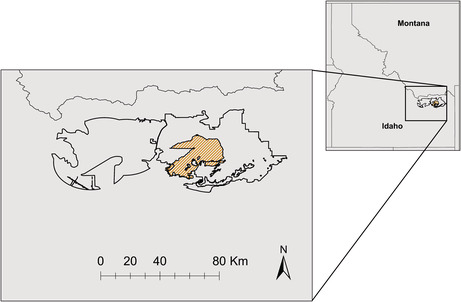

## 1 INTRODUCTION

Global change is fundamentally altering the nature of disturbance regimes, including the scale, severity, and frequency of disturbance events (Burton et al., 2020; Thom & Seidl, 2016; Turner, 2010). These changes have profound implications for plant and animal populations because they affect a broad array of processes, including genetic diversity and life history evolution (Banks et al., 2013; Lytle, 2001), biogeochemical pathways (Finzi et al., 2020), spread of invasive species (Bradley et al., 2009; Vitousek et al., 1996), and the population processes and species interactions that structure ecological communities (Dale et al., 2001; Jacquet & Altermatt, 2020; Peñuelas & Sardans, 2021). Consequently, large‐scale disturbance events structure the dynamics of vegetation and landcover, and thus biodiversity and the distribution and abundance of animals (Franklin et al., 2016; Hansen et al., 2001; Thom & Seidl, 2016).

Large‐scale wildfire events are becoming increasingly common and have far‐reaching implications for the conservation of habitat for terrestrial wildlife (Abatzoglou & Williams, 2016; Duane et al., 2021; McKenzie et al., 2003). Agents of global change that modify fire regimes include climate but also human‐induced modification of fuel structure (e.g., land use, fragmentation of vegetation) and patterns of ignition (Pausas & Keeley, 2021). Effects of global change on fire regimes vary regionally, but increases in the frequency of large fire events and the total area burned are widespread (Dennison et al., 2014; Ruffault et al., 2018; Seidl et al., 2011; Westerling et al., 2006). These changes pose both short‐ and long‐term challenges to the conservation of sensitive species (McKenzie et al., 2003) by shaping the composition and structure of postfire vegetation communities within terrestrial ecosystems (Franklin et al., 2016; Johnstone et al., 2010). For example, fires can shift species composition and alter successional dynamics, resulting in different plant communities that develop in a postfire landscpe (Johnstone et al., 2010; Wang et al., 2019).

Large‐scale disturbance events also shape the spatial and temporal distribution of high‐quality habitats for wildlife (Knopff et al., 2014; Moreau et al., 2012; Squires et al., 2020). By their very definition, pulse disturbances like fires result in immediate changes to the resources available to animals over broad landscapes (Burton et al., 2020; White & Pickett, 1985). Yet, few studies have directly assessed the degree to which megafire events (i.e., fires >10,000 ha; Linley et al., 2022) change space use patterns (i.e., use and selection of resources) of animals in habitat patches that remain. Functional responses in habitat use provide a general conceptual framework for understanding and predicting context‐dependent space use when patterns of use and selection change with resource availability (Holbrook et al., 2019; Matthiopoulos et al., 2011; Mysterud & Ims, 1998), but we are unaware of previous studies that assessed functional responses by sensitive species as a potential consequence of megafire. Habitat use and selection are fundamental to animal ecology and link animals with the communities and ecosystems they inhabit (Cagnacci et al., 2010). Therefore, failing to account for the effects of changes in resource availability induced by disturbance events may lead to misinformed predictions of habitat use in novel environments (Aarts et al., 2013; Holbrook et al., 2019), and thus have far‐reaching implications for conservation in the face of global change.

In the sagebrush steppe of western North America, fire poses a direct threat to the conservation of habitat for native wildlife (Coates et al., 2016; Connelly et al., 2011; Knick et al., 2003, 2011). Resilience of sagebrush‐steppe plant communities to fire is spatially heterogeneous and impacted by complicated interactions between a number of biotic and abiotic factors (e.g., species composition, moisture, elevation; Chambers et al., 2014; Davies et al., 2012; Ringos et al., 2019). For example, the invasion of exotic cheatgrass (*Bromus tectorum*) has facilitated changes in sagebrush‐steppe fire regimes, especially at lower elevations, increasing the frequency, severity, and extent of large fires (Baker, 2006; Balch et al., 2013; Whisenant, 1990). High‐severity fire kills most species of sagebrush (*Artemisia* spp.), thus changes to fire severity and frequency shift plant community composition in favor of more fire‐tolerant species (e.g., annual grasses). Changes to plant communities are also facilitated by larger fires, whereby conversion to grassland can result because sagebrush seedbanks in the soil are often minimal and seed dispersal distances are short (Reeves et al., 2018). Further, multiyear precipitation patterns affect the buildup of fine fuels in sagebrush steppe (from both native and non‐native herbs), which in turn affects the area burned and the probability of large fires (Pilliod et al., 2017). Even management efforts aimed at reducing fire risk in sagebrush steppe can change the composition of available habitat over large areas (e.g., fuel breaks; Shinneman et al., 2019). Not surprisingly, fire can also impact the composition of sagebrush‐steppe animal communities across a variety of taxa and trophic levels (Holbrook et al., 2016; Knick et al., 2003; Rhodes et al., 2010; Rohde et al., 2019). Understanding the responses of animal populations to large fires is critical for identifying conservation strategies that are likely to benefit sensitive species (Nimmo et al., 2021). Consequently, we need to assess potential changes in patterns of habitat use resulting from megafire in order to better predict responses of animal populations and conserve habitat for sensitive species in the face of global change.

Greater sage‐grouse (*Centrocercus urophasianus*; hereafter sage‐grouse) are a sagebrush obligate and an umbrella species for the conservation of sagebrush ecosystems (Rowland et al., 2006). Sage‐grouse populations have declined across the sagebrush biome (Coates et al., 2016, 2020; Garton et al., 2011), where the reduction of sagebrush cover has resulted in habitat loss and population extirpation (Aldridge et al., 2008; Wisdom et al., 2011). Fire can eliminate breeding habitat for sage‐grouse (Nelle et al., 2000; O'Neil et al., 2021; Rhodes et al., 2010) and reduce vital rates and populations over a variety of spatial scales (Blomberg et al., 2012; Connelly, Reese, et al., 2000; Dudley et al., 2021; Foster et al., 2019). Fire‐induced change in the distribution and quality of sage‐grouse habitat is well established, yet space use patterns may change over time in the aftermath of large fires as birds adapt to an altered landscape (Schuyler et al., 2022). Consequently, we studied space use by sage‐grouse before and after a high‐intensity megafire event that burned sagebrush steppe in eastern Idaho, USA, and addressed the dual goals of: (1) understanding how habitat use and selection change in response to changing resource availability induced by megafire, and (2) predicting the intensity of habitat selection in a post‐megafire landscape. We documented context‐dependent habitat use with changing environmental conditions, as well as the robustness of most functional response relationships across spatial scales. We also built resource selection functions (RSFs) to predict habitat selection as a function of changes to available vegetation and mapped the relative probability of selection before and after fire. Our results have strong implications for understanding the potential role of megafire in altering space use by animals in a changing environment.

## 2 MATERIALS AND METHODS

### 2.1 Study area

**FIGURE 1:**
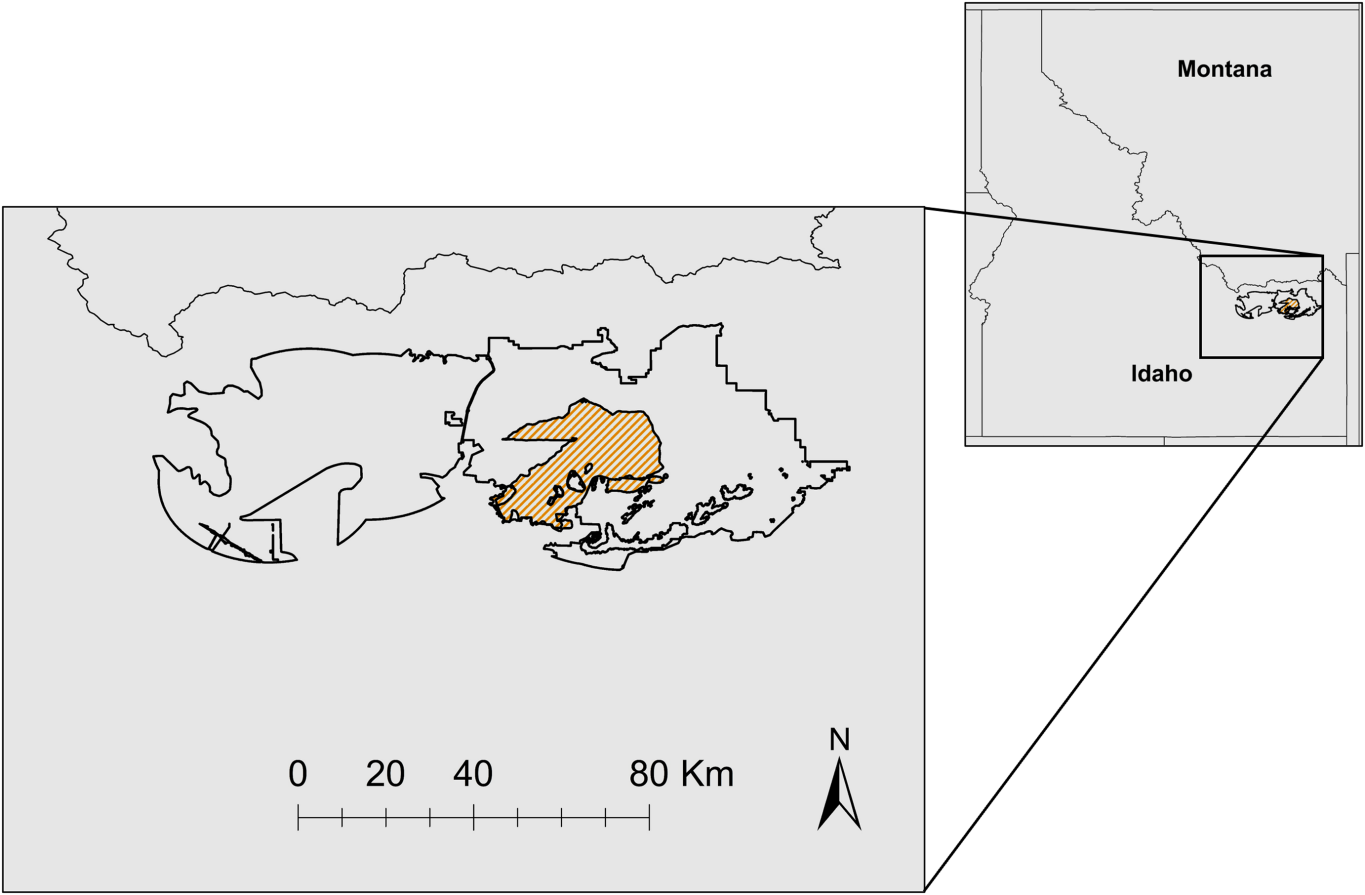
Map of study areas used to assess functional responses of greater sage‐grouse to catastrophic megafire in Eastern, Idaho, USA, during 2015–2020. Study site boundaries are indicated by the solid black lines, with Sand Creek on the right and Medicine Lodge on the left. The area burned by the Grassy Ridge fire is indicated by the orange hatching with a black outline on the Sand Creek site.

**FIGURE 2:**
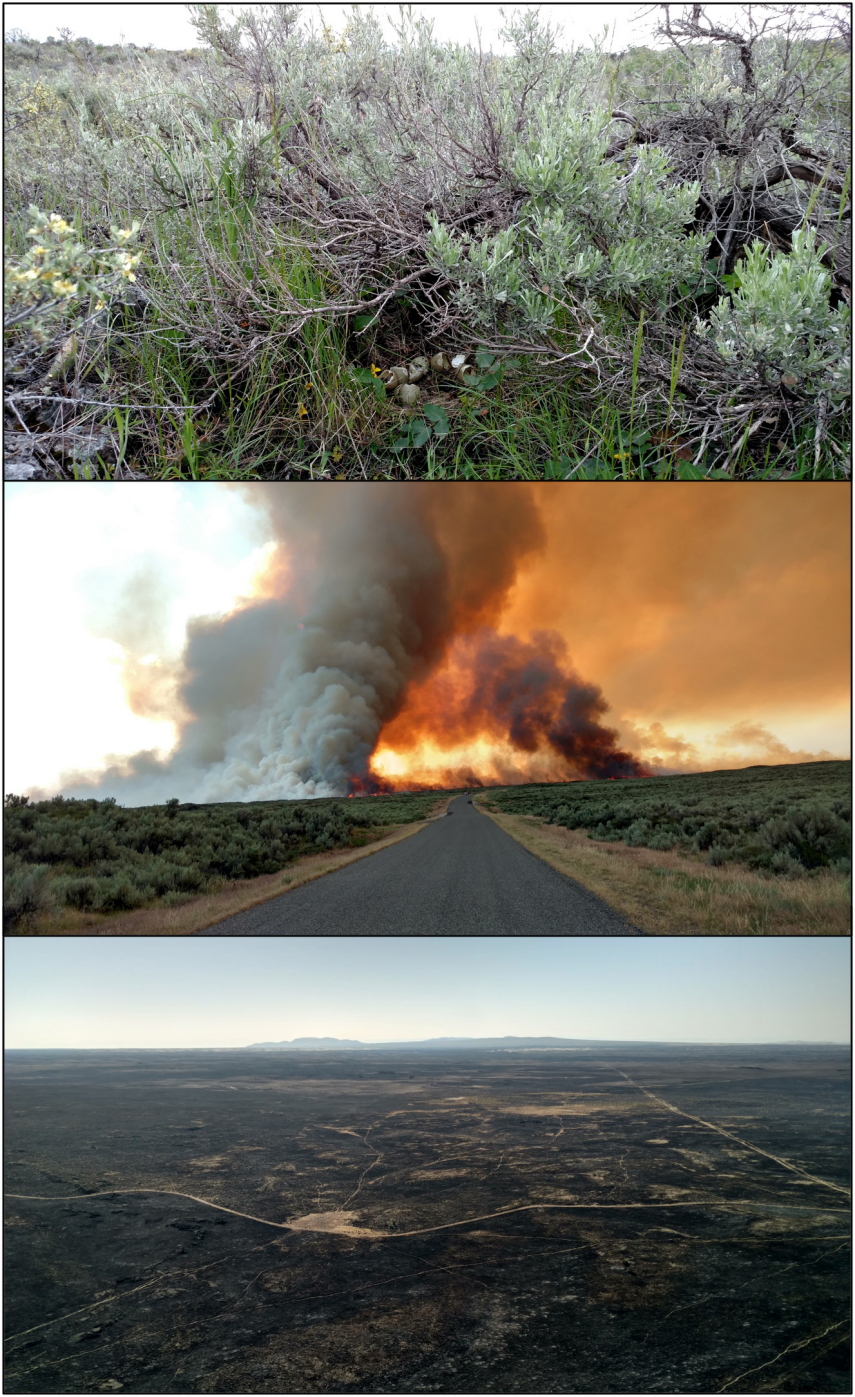
Pictures of a successful sage‐grouse nest (preburn) at the Sand Creek site with characteristic vegetation features (top), typical sagebrush‐steppe vegetation cover at Sand Creek with the Grassy Ridge Fire burning in the distance (middle), and an aerial view of the landscape directly after the fire, demonstrating the fire intensity (bottom). Photos were provided by D. Englestead.

We studied patterns of habitat use by sage‐grouse in high‐elevation sagebrush steppe on the Medicine Lodge and Sand Creek areas of eastern Idaho, USA (Figure 1). These study sites represent distinct breeding subpopulations of sage‐grouse; birds within each site have similar seasonal movements and nesting areas, but birds nesting at different sites may mix together during other periods of the year. Elevation at these sites ranged from approximately 1494 to 1951 m above sea level, and the areas were dominated by mesic, mountain big sagebrush (*Artemisia tridentata vaseyana*) plant communities. The Grassy Ridge Fire burned >40,000 ha of the Sand Creek study site in July–August 2018, thus qualifying as a megafire (>10,000 ha; Linley et al., 2022) and providing a natural experiment with the unburned Medicine Lodge site serving as a control. The Grassy Ridge Fire was also a high‐intensity burn that eliminated shrub cover and changed the composition of vegetation available to sage‐grouse across the region (Figure 2).

### 2.2 General approach

We monitored female sage‐grouse during six breeding seasons (2015–2020) and conducted analyses to understand and predict context‐dependent changes in habitat use as a function of changes to resource availability. We conducted population‐level analyses and assessed functional responses in both the use and selection of nest sites, as well functional responses in the use and selection of resources by females during brood rearing. For each of the three stages of the breeding season (nesting, early brood rearing, and late brood rearing), we used distinct analyses to: (1) document and test for context‐dependent habitat use in relation to changes in the availability of individual resources, and (2) predict resource selection before and after megafire while accounting for changes in resource availability through functional responses. These analyses were complimentary, evaluating habitat use and selection independently and considering both inferences about and prediction of space use. Therefore, these analyses furthered our understanding of the usefulness of functional responses as a framework for understanding and predicting the effects of megafire on patterns of habitat use and selection by sage‐grouse (Holbrook et al., 2017, 2019).

Inferences about habitat use can be sensitive to the observational scales used for measuring environmental conditions, and optimal scales at which animals select habitat features commonly vary among important covariates (DeCesare et al., 2012; McGarigal et al., 2016). Interpretation of functional responses may also be sensitive to elements of the scales used to measure habitat covariates (e.g., spatial grain; Laforge et al., 2016). Thus, we conducted multiscale assessments of habitat use while taking into account temporal and spatial heterogeneity in resource availability through functional responses. We measured habitat covariates over a range of relevant spatial scales, and: (1) assessed the robustness of our statistical inferences across measurement scales (see Understanding Context‐dependent Use), and (2) identified the optimal scale for each covariate for predicting resource selection (see Predicting Resource Selection).

### 2.3 Space use data

**FIGURE 3:**
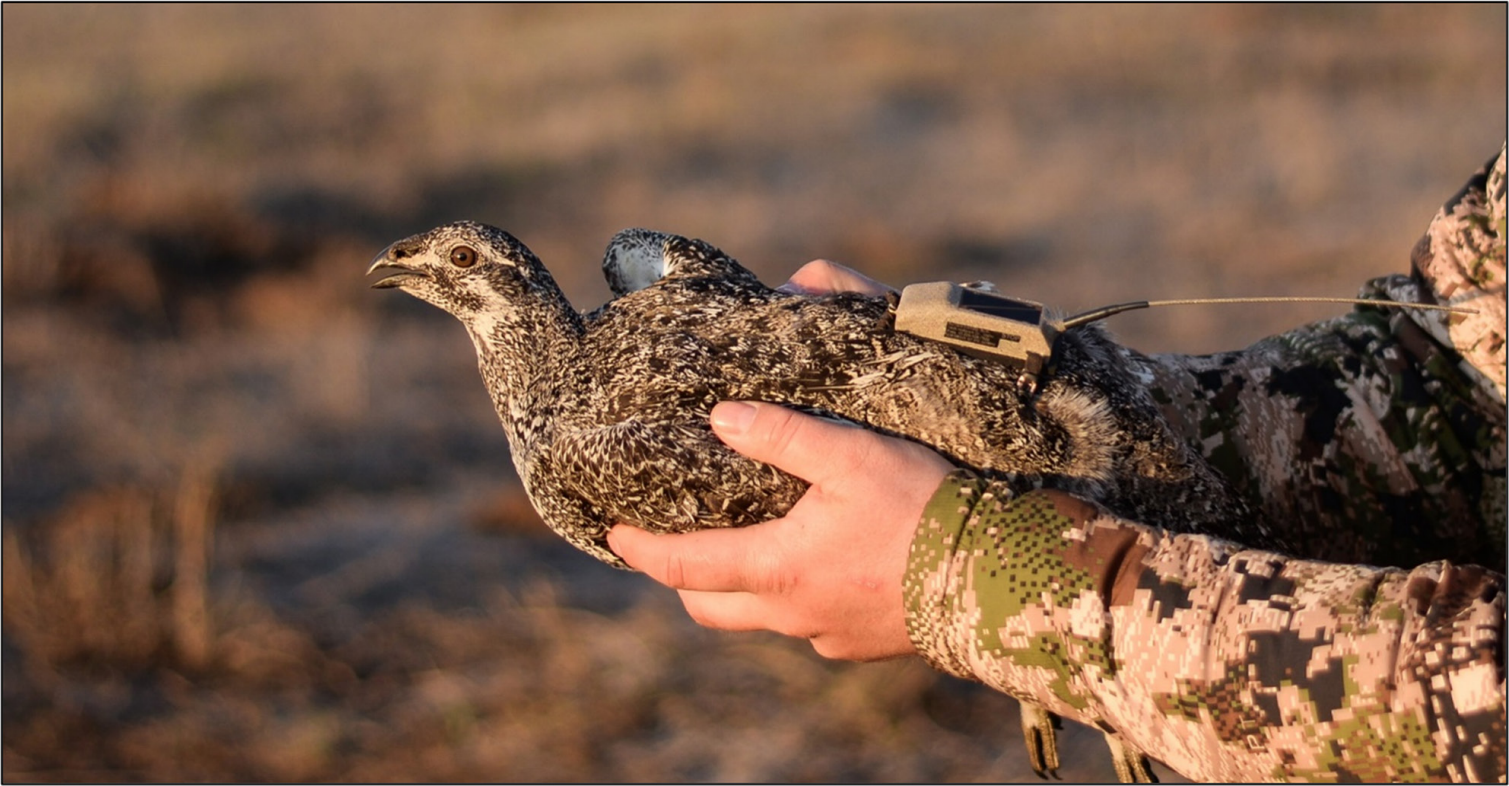
Picture of rump‐mounted, solar‐powered GPS platform terminal transmitter attached to female greater sage‐grouse in eastern Idaho, USA. Photo courtesy of D. Englestead.

We used standard spotlighting techniques (Wakkinen et al., 1992) or rocket nets to capture sage‐grouse on or near leks during the breeding season (March–April), fitted female birds with a rump‐mounted, solar‐powered GPS platform terminal transmitter (PTT; Model PTT‐100, Microwave Telemetry Inc.; Figure 3), and released birds at their capture locations (*n* = 269 nesting females from 2015 to 2020). We trapped and monitored female grouse at locations throughout each study area, from 2015 to 2020 at Sand Creek and from 2017 to 2020 at the Medicine Lodge site. We monitored female sage‐grouse daily to document breeding season space use. We programmed PTTs to collect six locations per day, with an upload frequency that depended on the season: more frequent data acquisition during nesting and early brood rearing and less frequent during other periods. We assumed that a female had initiated incubation at a nest (*n* = 309) when we recorded locations at the same coordinates for ≥3 days. We then added 26 days to the initiation date to predict the hatch date. When a female had several consecutive locations away from the nest site, we visited and inspected the nest to determine fate (i.e., loss or hatch) based on eggshell and nest‐site conditions in combination with estimated hatch timing. We documented nest fate so that we could identify females with broods.

We monitored the movements of all females, conducted flush surveys at 42 days posthatch (Riley et al., 2021; Riley & Conway, 2020) for females that hatched successful nests, and considered the brood successful if a female had at least one chick during the survey. We used PTT location data from females with successful broods to assess space use during the brood‐rearing period (*n* = 67 females with successful broods from 2015 to 2020). We limited our analyses to females with successful broods because the monitoring did not involve field‐intensive brood counts prior to the 42‐day flush surveys; thus, we did not know when unsuccessful broods were lost, and consequently had no way to distinguish brood use data from habitat use of the female after losing a brood. We split brood location data into early brood‐rearing (hatch to 21 days posthatch) and late brood‐rearing (22–42 days posthatch) because we believed space use may differ as hens with broods became more mobile and moved farther away from their nest sites. During the first 21 days posthatch, >95% of locations across all birds were <1.7 km from their nest site. We observed larger movements away from nests (>1.7 km) for most females with broods (62.7%), and these movements usually began on or after 21 days (79.1% of females with broods). However, we visually evaluated location data and defined this cutoff between early and late periods differently for birds that began directional movements away from nests earlier than 21 days posthatch. Specifically, we adjusted the cutoff earlier for females that clearly began to move out of nesting areas (i.e., >1.7 km from the nest) with broods prior to 21 days posthatch. We adjusted the early‐late brood‐rearing cutoff for 21.9% of females with broods (range = 5–20 days). This resulted in 7239 and 9442 used locations from 67 females with broods across the early and late periods, respectively.

### 2.4 Environmental data

We measured vegetation at used and available locations to assess functional responses in the use and selection of resources resulting from changes to available habitat. We assessed the selection of nest sites within the landscape and consequently defined available conditions for nesting as located in sage‐grouse habitat within 18 km of locations where birds were trapped (Makela & Major, 2012; priority and general habitat). Within this region, we used *spsurvey* (Dumelle et al., 2021) in R version 4.0.1 (R Core Team, 2017) to generate 500 spatially balanced random points to measure available resources for nesting. We also assessed selection of early and late brood‐rearing habitat by females with broods. For these analyses, we defined available early brood‐rearing habitat for each bird as areas located within 1.7 km of the nest, because a 1.7 km buffer contained >95% of early brood‐rearing locations across all birds. Similarly, we defined available late brood‐rearing habitat for each bird as areas located within 8 km of the nest, a buffer that contained >95% of late brood‐rearing locations. We generated a 10:1 ratio (Barbet‐Massin et al., 2012; Northrup et al., 2013) of random points to use points for each female to measure available resources during the brood periods. Environmental data were collected at fewer random points for nesting because sampling these points was considerably more labor intensive, as they were visited in the field to conduct vegetation measurements (described below).

We measured microsite vegetation characteristics in the field at nest sites within 1 week of nest fate, and random points between 1 May and 30 June each year (corresponding to the earliest and latest nest fates). We used the Bureau of Land Management (BLM) habitat assessment framework (HAF) with integrated terrestrial assessment, inventory, and monitoring (AIM) vegetation sampling protocol (hereafter HAF‐AIM) to measure microsite vegetation characteristics (Lepak et al., 2018), including the following variables that we predicted would influence nest‐site selection based on prior studies (e.g., Smith et al., 2020): sagebrush cover, total shrub cover, sagebrush height, perennial grass cover, perennial grass height, perennial forb height, forb cover, forb height, and forb richness. A single, 50‐m HAF‐AIM transect (hereafter transect) was centered on the nest bowl (or random point) and either ran in a consistent (e.g., north) or random direction.

We used the remotely sensed Rangeland, Condition, Monitoring, Assessment, and Projection (RCMAP) fractional component time series data (Rigge et al., 2019, Rigge et al., 2020; accessed 7/2020) to measure vegetation characteristics at a variety of spatial extents around used and available locations for both nesting and brood‐rearing analyses. The RCMAP raster data provided annual cover measures at a 30‐m resolution across rangelands of the western United States and thus provided a relatively fine‐resolution snapshot of resources available to greater sage‐grouse each year. The pixel‐scale cover predictions from RCMAP are based on Landsat imagery from three periods each year (spring, summer, fall), but these data are augmented with additional independent data (e.g., slope, aspect, field measurements, high resolution [2 m] satellite imagery, fire perimeter data, etc.; Rigge et al., 2020) inside a machine learning algorithm to generate cover predictions. Moreover, for fire events that occur within a mapping year (e.g., the Grassy Ridge Fire in 2018), seasonal imagery capturing postfire conditions are used for prediction to ensure that the effects of recent burns on vegetation are captured (Rigge et al., 2020). We used the following continuous cover rasters from the RCMAP data set: sagebrush cover, shrub cover, herbaceous cover, annual herbaceous cover, litter cover, and cover of bare ground. We also derived a raster for perennial herbaceous cover by subtracting the annual herbaceous cover from the total herbaceous cover at the pixel scale. The RCMAP data provided annual cover rasters from 1984 to 2018 and thus did not cover the final 2 years of our study (2019–2020). However, the 2018 RCMAP data captured the effects of the Grassy Ridge Fire on vegetation, and thus described large‐scale changes to the resources available to nesting and brood‐rearing sage‐grouse directly after the fire. Thus, we used 2018 RCMAP data to measure remotely sensed vegetation characteristics at used and available points during 2019–2020. We also characterized sagebrush height from the RCMAP sagebrush height raster; however, sagebrush height was not available annually, and consequently, we used the data year closest to our study (2015). From the 2015 sagebrush height raster, we derived a postfire raster of height by setting pixels contained within the fire perimeter to zero, because the high‐intensity fire resulted in little standing live sagebrush (Figure 2).

All remotely sensed vegetation metrics were measured over a range of spatial extents using moving window analyses (100–1000 m radii, in 100 m increments). For example, sagebrush cover at the 100 m extent was calculated for a given 30‐m pixel by averaging continuous cover values over all raster pixels within 100 m; moving window analyses replicated this process for each pixel within a raster. Similarly, this process was replicated for each spatial extent and for each vegetation metric. We also derived a raster to measure the spatial heterogeneity of sagebrush cover across the same range of spatial extents. First, we reclassified pixel‐scale continuous sagebrush cover values into biologically relevant (Connelly, Schroeder, et al., 2000) cover categories (≤5%, 6%–15%, 16%–25%, 26%–35%, 36%–45%, and >45%). We then used moving window analyses to calculate Simpson's diversity index on the categorized cover raster. We replicated this across all spatial extents to capture multiscale changes in the heterogeneity of sagebrush cover.

Lastly, we measured covariates that we expected could influence sage‐grouse space use to include as control variables in predictive habitat models (see Predicting Resource Selection below). Specifically, we measured the distance from each nest site and random point to the nearest sage‐grouse lek (included in nesting models only), distance from the nearest mesic habitat patch (included both mesic grass and mesic shrub habitat; nesting models) or mesic grass habitat patch (brood habitat models), topographic roughness (Riley et al., 1999), an index of wetness (compound topographic index [CTI]; Gessler et al., 1995, Moore et al., 1993), and a measure of sun exposure (site exposure index; Balice et al., 2000). Topographic roughness, CTI, and the site exposure index were all calculated from a 10‐m digital elevation model at the point location of interest (use and random points), whereas mesic and mesic grass habitat patches were obtained from a fine‐resolution (1‐m) vegetative cover raster recently created for the state of Idaho (resampled to 30‐m resolution; Idaho Department of Fish and Game, unpublished data). The fine‐resolution vegetative cover raster used supervised classification of 2014 National Agriculture Imagery Program (NAIP; USDA Farm Service Agency) imagery to map vegetation cover across the state of Idaho. Lastly, we included variables related to the fire location, in order to capture any direct avoidance of the fire scar by nesting and brood‐rearing sage‐grouse: continuous distance outside of the fire perimeter (i.e., negative values for locations inside the burn scar; 2019–2020 data and brood models only, where covariate was treated as missing [zero inserted after standardization] for data prior to 2019), and a binary variable to indicate whether a location was inside the fire perimeter (one for 2019–2020 points inside the perimeter, zero otherwise; nesting models only). We conducted all spatial analyses with ArcMap 10.5.1 (ESRI). Moreover, we used the Geomorphometric and Gradient Metrics toolbox (version 2.0) in ArcMap to calculate topographic roughness, CTI, and the site exposure index.

### 2.5 Understanding context‐dependent use

**FIGURE 4:**
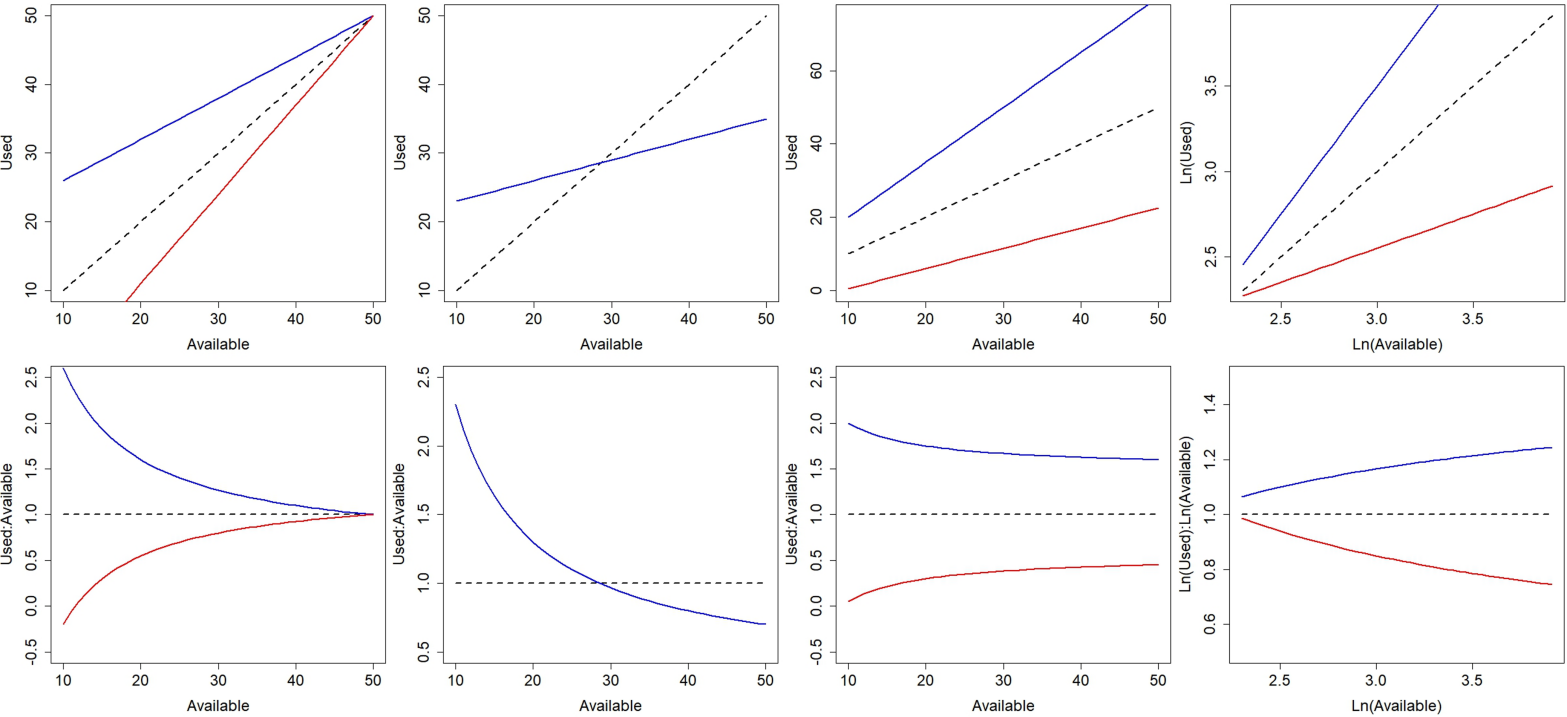
Conceptual diagrams of hypothetical functional response relationships for additive (top row, used vs. available) and multiplicative (bottom row, used: available ratio vs. available) scale definitions of habitat use. Hypothetical functional responses shown are (left to right): relaxed selection (blue) and avoidance (red), tradeoffs, increased (blue) and decreased (red) use, and increased (blue) and decreased (red) selection. Increased and decreased use are conceptually and mathematically different than increased and decreased selection, respectively. Consequently, increased and decreased use must be assessed on the additive scale, whereas increased and decreased selection are assessed on the natural log scale (Holbrook et al., 2019). Dashed lines represent a 1:1 relationship between used and available habitat and hence proportional use with no selection. Verbal descriptions of each functional response portrayed here are provided in Table 1.

We conducted univariate analyses to explicitly test for context‐dependent use of vegetation by female sage‐grouse in response to changing environmental conditions. Specifically, we conducted approach 1 and approach 2 analyses (described by Holbrook et al., 2019) to assess functional responses in the use and selection of resources during nesting and brood rearing. Approach 1 analyses used linear regression to regress average values at use locations against average values at available locations, individually for each covariate, and tested the null hypothesis of no context dependence in use of resources on an additive scale. Approach 2 analyses used linear regression to regress the natural log of average values at use locations against the natural log of average values at available locations and tested the null hypothesis of no context dependence in use of resources on a multiplicative scale, which is equivalent to testing for context dependence in selection of resources (i.e., use‐to‐available ratios; Holbrook et al., 2019). Thus, analysis approaches 1 and 2 tested complementary hypotheses for context‐dependent habitat use and selection, where statistical results and their ecological interpretation can differ between approaches (Tables 1 and 2; Figure 4). That is, context‐dependent use can be present without context‐dependent selection, and vice versa, and changing the scale of assessment changes the hypothesis being tested and the interpretation of results.

**TABLE 1:** Categories of functional responses and their ecological and graphical descriptions, for observed responses that were relevant to greater sage‐grouse in eastern Idaho, USA, 2015–2020.

Functional response category^a^	Description
Relaxed	Resource is selected or avoided when scarce (low availability), but the strength of selection or avoidance decreases as the resource becomes more abundant (availability increases). Graphical relationships between used and available resources approach the 1:1 line representing proportional use
Tradeoff	Resource is selected when scarce (low availability) but becomes avoided as the resource becomes more abundant (availability increases). The graphical relationship between used and available resources begins above but then crosses to be below the 1:1 line representing proportional use
Increased or decreased use	Resource use is greater than (increased) or less than (decreased) proportional use, but the absolute difference between used and available resources increases as the resource becomes more abundant (availability increases). Graphical relationships between used and available resources get progressively farther from the 1:1 line representing proportional use on the real scale
Increased or decreased selection	Resource is selected (increased) or avoided (decreased), but the strength of selection or avoidance increases as the resource becomes more abundant (availability increases). Graphical relationships between used and available resources get progressively farther from the 1:1 line representing proportional use on the natural log scale

^a^ Additional details about functional response scenarios, and examples of each, are reviewed by Holbrook et al. (2019).

**TABLE 2:** Parameter combinations that were used to interpret and infer functional responses in additive (approach 1) and multiplicative (approach 2) scale definitions of habitat use for nesting and brood‐rearing greater sage‐grouse in eastern Idaho, USA, 2015–2020.

Analysis approach and interpretation^a^	$\beta_{0}$	$\beta_{1}$
Approach 1		
Proportional use	0	1
Additive use	≠0	1
Increased use	>0	>1
Relaxed or tradeoff	>0	<1
Relaxed	<0	>1
Decreased use	<0	<1
Approach 2		
Proportional selection^b^	0	1
Additive selection^c^	≠0	1
Increased selection	>0	>1
Relaxed or tradeoff	>0	<1
Relaxed	<0	>1
Decreased selection, tradeoff, or relaxed	<0	<1

*Note*: These parameters represent the intercept ($\beta_{0}$) and coefficient ($\beta_{1}$) terms from linear regression analyses of average used vs. average available conditions (approach 1) and ln(average used) vs. ln(average available) conditions (approach 2) and were used in tandem with graphical plots to interpret functional responses (Table 1, Figure 4), or lack thereof (i.e., additive or proportional use).

^a^ Under approach 1, $\beta_{1}$ > 1 implies that the use of resources is increasing relative to random, whereas $\beta_{1}$ < 1 implies use is decreasing relative to random. Under approach 2, $\beta_{1}$ > 1 implies the ratio of used: available resources is increasing relative to random (i.e., strength of selection is increasing), whereas $\beta_{1}$ < 1 implies the ratio of used: available resources is decreasing relative to random.

^b^ This is equivalent to no selection for or against a resource.

^c^ This is equivalent to a constant selection ratio that does not change with availability.

A primary motivation in testing for context‐dependent habitat use is to understand changes in the behavioral patterns of habitat use in response to changing resource availability (see Holbrook et al., 2019 and citations therein), whereas selection is itself calculated as a mathematical function of resource availability (i.e., use‐to‐available ratio). Thus, functional responses in selection can be an artifact of changing available conditions even when patterns of habitat use remain unchanged (Beyer et al., 2010). Context‐dependent use is therefore more directly indicative of changes to spatial patterns of behavior with changing resources, and performing approach 1 and 2 analyses in tandem is a conservative approach to inferring how space use responds to changing environmental conditions (Holbrook et al., 2019).

Under the null hypothesis of proportional use, deviations from the null hypothesis are indicated by statistical tests on the values of the regression coefficients. For analysis approaches 1 and 2, proportional use or selection occurs when $\beta_{0}$ = 0 and $\beta_{1}$ = 1, whereas disproportionate use or selection occurs otherwise (Holbrook et al., 2017). When $\beta_{0}$ ≠ 0 habitat use is different from available conditions when resource availability is low, and when this occurs and $\beta_{1}$ = 1 the additive difference between used and available habitat is constant for approach 1, whereas the ratio of used to available habitat values is constant for approach 2 (i.e., additive use or selection). When $\beta_{1}$ ≠ 1 resource use changes in a nonrandom fashion as resource availability changes, hence the additive difference between used and available habitat (approach 1) or the selection ratio (approach 2) is not constant, providing evidence for a functional response (Tables 1 and 2; Figure 4). The combination of $\beta_{0}$ and $\beta_{1}$ values can therefore indicate the type of response observed (Tables 1 and 2), yet the ecological interpretation also requires graphical evaluation (Figure 4) because the combinations of $\beta_{0}$ and $\beta_{1}$ values produced by different functional responses are not mutually exclusive (Table 1). We tested for context‐dependent habitat use for all covariates, yet sample units for these analyses differed among periods of the breeding season. The sample unit was the study area‐year combination (*n* = 9) to test for functional responses in the use and selection of nest sites (i.e., averages were taken over used and available locations for site‐year combinations), whereas the brood (*n* = 67 broods) was the sample unit for brood analyses (i.e., averages were taken over each individual brood). Analysis approaches 1 and 2 were also replicated over each spatial extent to assess the robustness of our inferences across scales.

### 2.6 Predicting resource selection

The utility of RSFs for conservation and management purposes is the creation of mapped predictions, thus we developed RSFs using model selection techniques that explicitly optimize model complexity for out‐of‐sample prediction (Gerber & Northrup, 2019). We developed population‐level, multiscale RSFs to predict resource selection by sage‐grouse with generalized functional response (GFR) models (Matthiopoulos et al., 2011), which provide a flexible statistical approach for predicting the relative probability of selection in space while accounting for changes in resource availability (approach 4 described by Holbrook et al., 2019). These models included functional responses through interaction terms between covariate values observed at individual use and available locations with the average values observed across available locations, where average available conditions were measured as described above (site‐year combination for nesting, individual for brood rearing).

We used infinitely weighted logistic regression to estimate RSF selection coefficients and used continuous model selection via LASSO regression to optimize model complexity for out‐of‐sample prediction (Gerber & Northrup, 2019). Specifically, we optimized the regularization parameter (λ) that dictates the inclusion and shrinkage of individual regression coefficients for out‐of‐sample prediction by using 10‐fold cross‐validation, with prediction performance measured via the average value of the area under the receiver operating curve calculated across holdout cross‐validation folds (test AUC). This effectively merged model selection and validation into a single process for the specific objective of predictive modeling (e.g., Gerber & Northrup, 2019; Stevens & Conway, 2019). We conducted all analyses with the *glmnet* package (Friedman et al., 2010) in R version 4.0.1. While our primary goal for RSF modeling was prediction of habitat selection, we recognize that predictive model selection may not generate the same ecological inferences from resource selection functions as models selected for inferential purposes (e.g., using information criteria; Gerber & Northrup, 2019). Thus, inferential analyses described above combined with predictive RSF modeling described here were complimentary, allowing us to directly test hypothesized functional responses for individual components of habitat while also mapping changes to the relative probability of selection under changing environmental conditions for management and conservation purposes (Gerber & Northrup, 2019; Holbrook et al., 2019).

We used a multi‐step process to arrive at a final RSF model. For the first step, we conducted univariate analyses to optimize the scale (100–1000 m) for each remotely sensed vegetation covariate, and to evaluate support for quadratic terms and functional response interactions for quadratic terms (brood models only) in the final model (i.e., $\beta \left(x_{i}^{2}\times {\bar{A}}_{x}\right)$, where $x_{i}$ is the value of covariate *x* at location *i* and ${\bar{A}}_{x}$ is the average value of *x* across available locations). For nest selection analyses, we fit models that included linear and quadratic main effects and the 1st‐order functional response interaction (i.e., $\beta \left(x_{i}\times {\bar{A}}_{x}\right)$), whereas larger sample sizes allowed us to also include functional response interactions for quadratic terms (i.e., $\beta \left(x_{i}^{2}\times {\bar{A}}_{x}\right)$) for brood selection analyses. For each covariate, we selected the shape (linear vs. quadratic) and scale (100–1000 m) to optimize out‐of‐sample prediction, as described above. Preliminary analyses suggested unrealistic results with quadratic terms for some covariates due to the sparsity of data across portions of the observed data range. Thus, we did not consider quadratic terms for annual herbs, litter, or bare ground in our nest‐selection RSFs. Similarly, we did not consider functional response interactions with quadratic terms for annual herbs, perennial herbs, and litter in our brood selection RSFs.

After identifying the optimal scale and shape through univariate analyses, we built multivariate and multiscale RSFs. For this final step, we included in our global model each remotely sensed variable at its optimal scale and shape, included first‐order functional response interactions ($\beta \left(x_{i}\times {\bar{A}}_{x}\right)$) for all covariates, and included second‐order functional response interactions ($\beta \left(x_{i}^{2}\times {\bar{A}}_{x}\right)$) for brood models when supported by univariate analyses. However, several variables were excluded from the global model because of strong correlation with variables that had better performance in univariate analyses (all models: total shrub cover and sagebrush height were strongly correlated with sagebrush cover; brood models: herbaceous cover was strongly correlated with correlated with perennial herbaceous cover). For transect‐scale variables in nesting RSFs, we included a fixed effect for each variable and its functional response interaction in the global model, as preliminary analyses found no support for quadratic relationships. We conducted model selection from the global model and used LASSO regression to identify the final model complexity (i.e., covariate inclusion and parameter estimates).

We conducted additional sensitivity analyses to evaluate the implications of autocorrelation for our RSF models, and also the transferability of habitat selection relationships and mapped predictions for models built using only data collected prior to the fire. To evaluate the implications of temporal autocorrelation for fitted selection coefficients, we used data thinning and re‐fit the final RSF models for early and late brood selection after randomly selecting only 3 use points per day per female (consistent with the low‐intensity sampling scenario of Gerber & Northrup, 2019). To evaluate the ability of models built prior to a large fire to predict space use in the same landscape post‐megafire (i.e., the temporal transferability of RSF predictions built using prefire data), we re‐fit the optimal RSF models using only data collected prior to the Grassy Ridge Fire (both including and excluding the functional response interaction terms). This provided a more detailed understanding of the sensitivity of selection relationships and model predictions to the observed range of available environmental conditions (i.e., smaller range for habitat covariates using only prefire data), as well as the ability of models built using prefire data to predict habitat use in a post‐megafire landscape.

We next developed raster models of RSFs to map the relative probability of selection across our study sites both before and after the Grassy Ridge Fire. This allowed us to document spatial shifts in the distribution of key breeding habitat induced by the fire, which can be used for habitat management and conservation planning purposes. We started with rasters of each scale‐optimized variable included in the final RSF for each of the three stages of the breeding season (nesting, early brood rearing, and late brood rearing). We then created rasters of average available conditions (before fire [2017] and after fire [2018]) for each covariate whose functional response terms were included in the final RSFs. For nest‐selection RSFs, the average available habitat for each covariate was again defined using site‐year combinations; thus, for each site (Medicine Lodge and Sand Creek) and year (2017 and 2018) combination, each 30‐m pixel was given the same value ($\bar{x}$ over all pixels) for average available habitat. However, our sampling of available habitat for brood selection was conditioned on nest locations. Thus, the average available habitat for each covariate in brood selection RSFs was mapped at a 30‐m resolution via moving window analyses, with window sizes of 1.7 km (early brood‐rearing period) and 8 km (late brood‐rearing period), respectively. We exponentiated the final RSFs to map the pixel‐scale relative probability of selection for nesting, early brood‐rearing, and late brood‐rearing periods. Because few females with broods ventured >500 m inside the boundary of the fire, we also manually set areas >500 m inside the fire perimeter to zero for the early and late brood maps, as these areas were effectively unavailable (i.e., non‐habitat). We also generated a composite RSF that aggregated final model predictions across nesting and brood‐rearing periods by multiplying the three rasters, effectively weighting the brood locations by the probability of a site being used for nesting (Appendix A). Finally, we replicated the mapping process for nest‐site and brood‐rearing models using RSFs fit only to prefire data, projecting these RSF predictions onto the post‐megafire landscape. This allowed us to further understand the temporal transferability of mapped predictions generated using only prefire data and the accuracy of such maps for postfire habitat management and conservation planning purposes.

Lastly, to visually portray the relative probability of selection for all models and evaluate the fit of mapped predictions, we reclassified continuous predictions into 10 ordinal categories representing low (1) to high (10) use (Boyce et al., 2002). We sampled predicted values for each of the final RSFs (nesting, early and late brood rearing) mapped to prefire conditions with 100,000 random points and used deciles (i.e., every 10th percentile) of the distribution of predicted values to set the category boundaries for each mapped RSF. We retained the same category boundaries prefire and postfire for each RSF, which allowed us to visually assess temporal changes in predicted selection relative to prefire conditions. Finally, we assessed the fit of mapped RSFs to the observed use data. We assigned each observed use point to the predicted ordinal category (1–10) of each map based on location and used Spearman's rank correlation to test for correlation between the category value and frequency of observed use points (Boyce et al., 2002). We used ArcMap 10.5.1 for all spatial RSF mapping and R version 4.0.1 to assess the fit of RSF maps to the observed use data.

## 3 RESULTS

### 3.1 Understanding context‐dependent use

**FIGURE 5:**
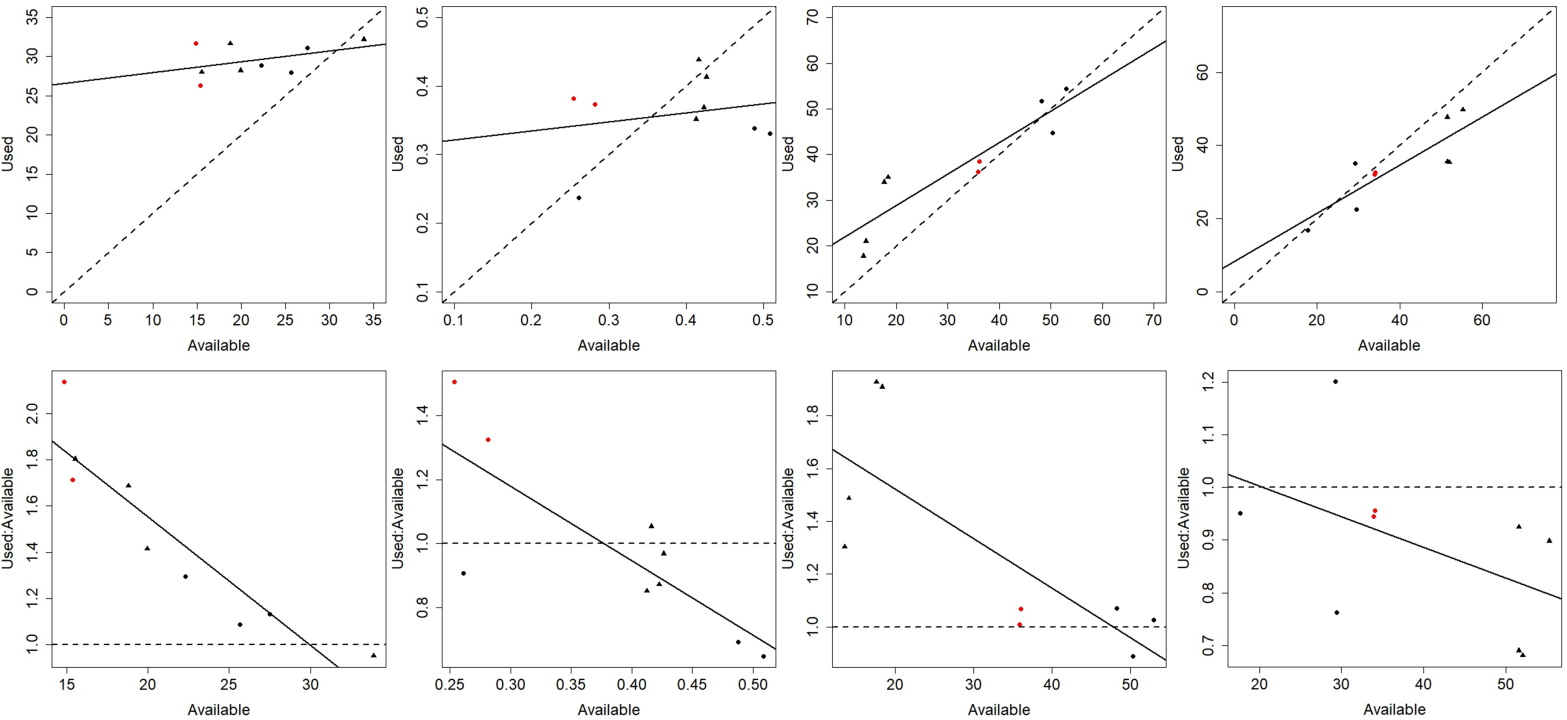
Univariate functional response relationships observed from greater sage‐grouse nest‐site selection analyses for eastern Idaho, USA, during 2015–2020. Shown are results from additive scale habitat use analyses (approach 1) regressing average used conditions against average available conditions (top row) for the following variables (left to right): percent sagebrush cover (transect scale), diversity of sagebrush cover classes (1000 m scale), sagebrush height (1000 m scale), and percent cover of bare ground (1000 m scale). Also shown are the multiplicative scale habitat use relationships (bottom row), with used: available habitat (i.e., selection ratios) regressed against average available conditions for each variable. Dashed lines represent a 1:1 relationship between used and available habitat for each variable and hence proportional use. Interpretation of each functional response is provided in Table 3.

**FIGURE 6:**
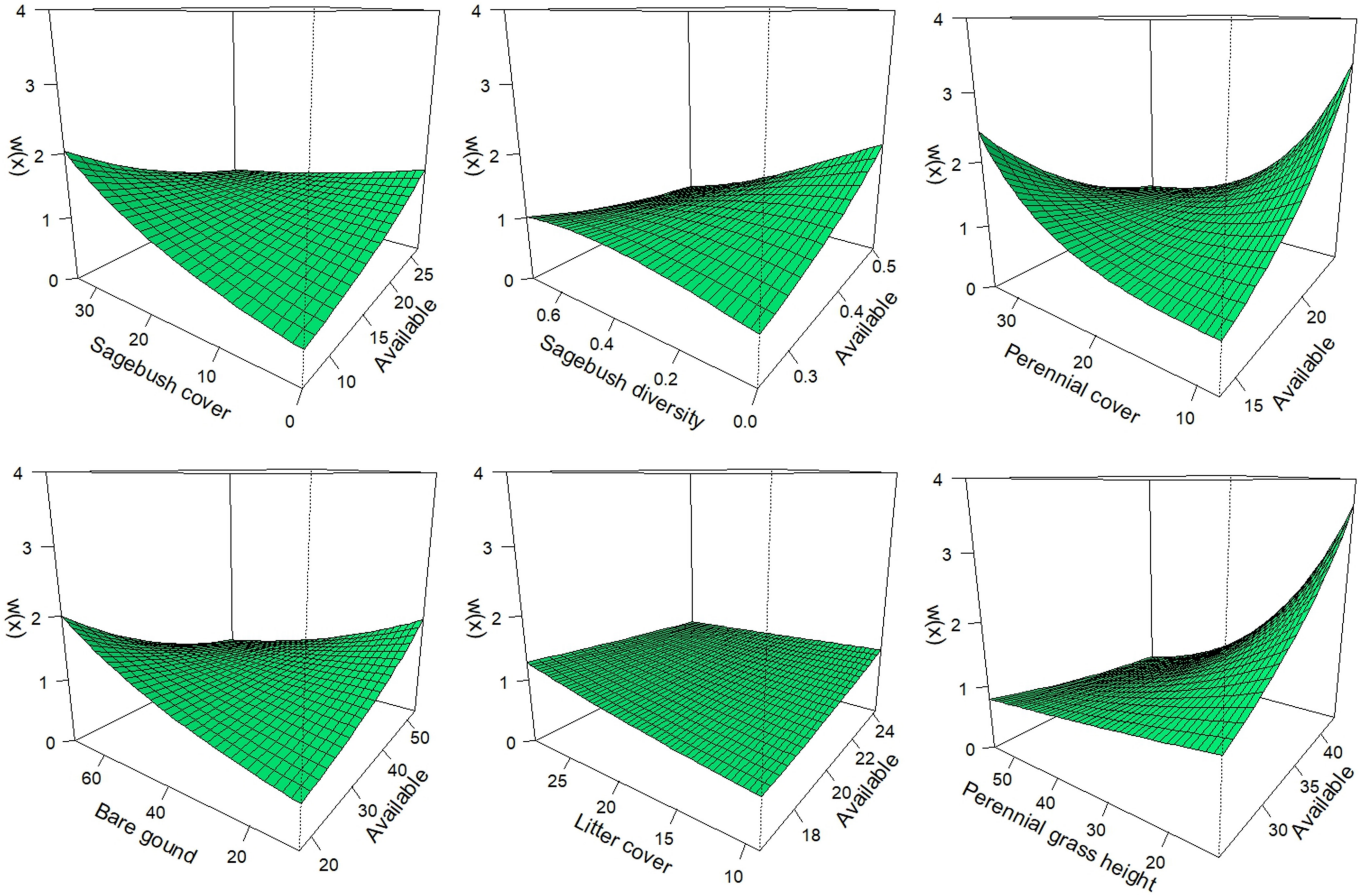
Functional response interactions observed for the resource selection function (RSF) developed for nest‐site selection by greater sage‐grouse in eastern Idaho, USA, during 2015–2020. Results are interactions from the final RSF (analysis approach 4) for percent sagebrush cover (100 m scale; top left), diversity of sagebrush cover categories (1000 m scale; top middle), percent cover of perennial herbs (500 m scale; top right), percent cover of bare ground (1000 m scale; bottom left), percent cover of litter (1000 m scale; bottom middle), and perennial grass height (transect scale; bottom right). The response variable w represents the relative probability of use. Interpretation of functional response interactions is Table 3.

**FIGURE 7:**
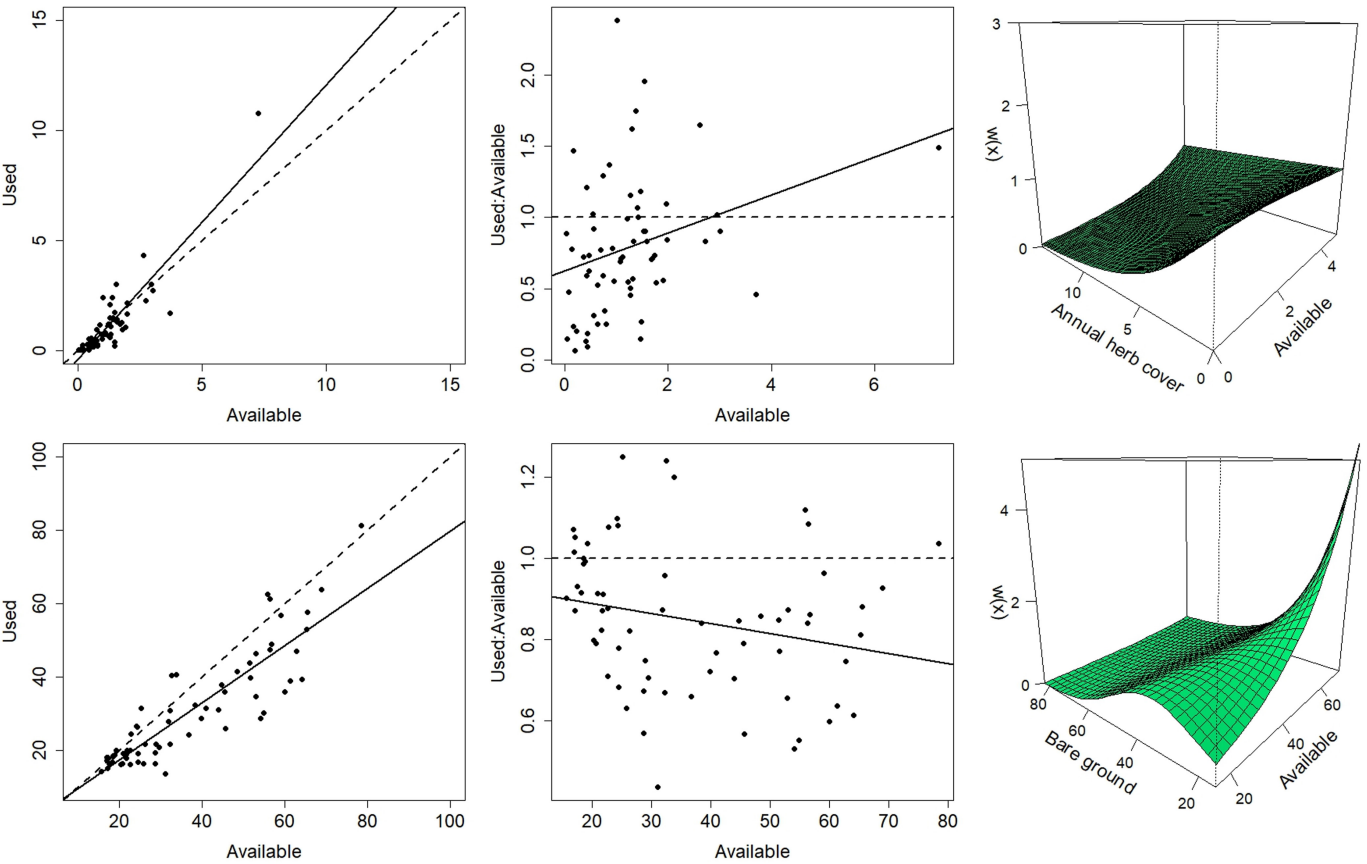
Example functional responses observed from greater sage‐grouse brood habitat selection for eastern Idaho, USA, during 2015–2020. Results are presented for additive scale (approach 1; left column; average used cover vs. average available cover) and multiplicative scale (approach 2; middle column; ln(average used cover) vs. ln(average available cover)) definitions of habitat use, as well as for the resource selection functions interaction terms (approach 4; right column; w is relative probability of use), for percent cover of annual herbs (early brood period, top row; 500 m scale for all plots) and percent cover of bare ground (late brood period, bottom row; 300 m scale for all plots).

**FIGURE A1:**
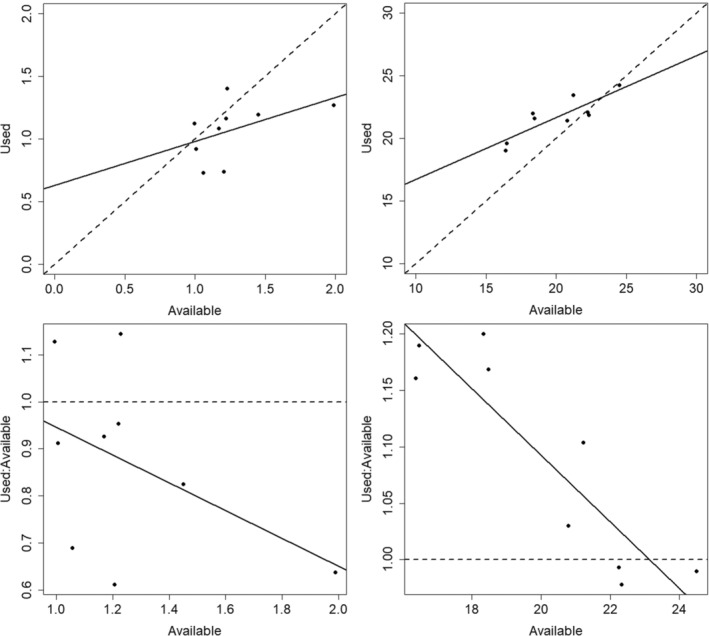
Additional univariate functional response relationships observed from greater sage‐grouse nest‐site selection analyses for eastern Idaho, USA, during 2015–2020. Shown are results from additive scale habitat use analyses (approach 1) regressing average used conditions against average available conditions (top row) for the following remotely sensed variables: percent cover of annual herbs (400 m scale; left), and litter cover (1000 m scale; right). Also shown are the multiplicative scale habitat use relationships (bottom row), with used: available habitat (i.e., selection ratios) regressed against average available conditions for each variable. Dashed lines represent a 1:1 relationship between used and available habitat for each variable and hence proportional use. Interpretations of functional responses are provided in Table 3.

**FIGURE A2:**
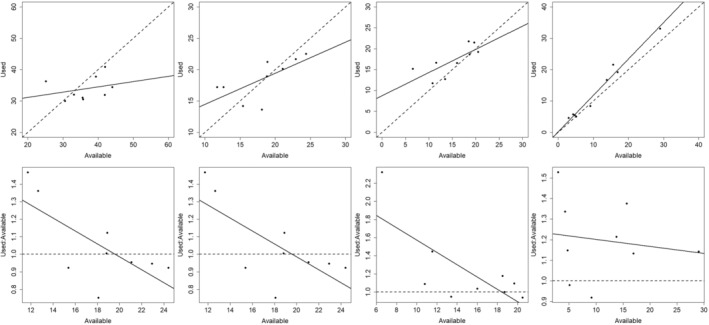
Additional univariate functional response relationships observed from greater sage‐grouse nest‐site selection analyses for eastern Idaho, USA, during 2015–2020. Shown are results from additive scale habitat use analyses (approach 1) regressing average used conditions against average available conditions (top row) for the following variables measured in the field at the transect scale (left to right): perennial grass height, perennial forb height, forb cover, and forb richness. Also shown are the multiplicative scale habitat use relationships (bottom row), with used: available habitat (i.e., selection ratios) regressed against average available conditions for each variable. Dashed lines represent a 1:1 relationship between used and available habitat for each variable and hence proportional use. Ecological interpretations of functional responses are provided in Table 3.

**FIGURE A3:**
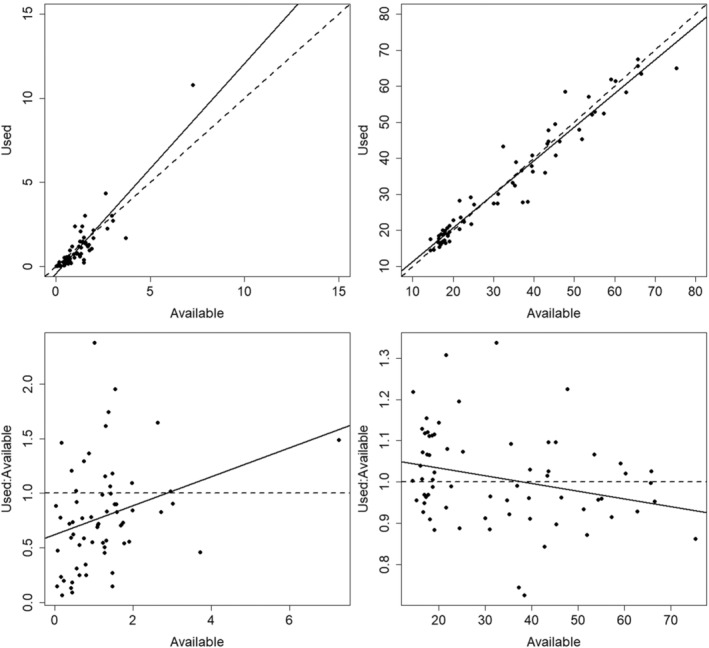
Additional univariate functional response relationships observed from greater sage‐grouse early brood selection analyses for eastern Idaho, USA, during 2015–2020. Shown are results from additive scale habitat use analyses (approach 1) regressing average used conditions against average available conditions (top row) for the following variables: percent cover of annual herbs (500 m scale; left), and percent cover of bare ground (100 m scale; right). Also shown are the multiplicative scale habitat use relationships (bottom row), with used: available habitat (i.e., selection ratios) regressed against average available conditions for each variable. Dashed lines represent a 1:1 relationship between used and available habitat for each variable and hence proportional use. Ecological interpretations of functional responses are provided in Table 4.

**FIGURE A4:**
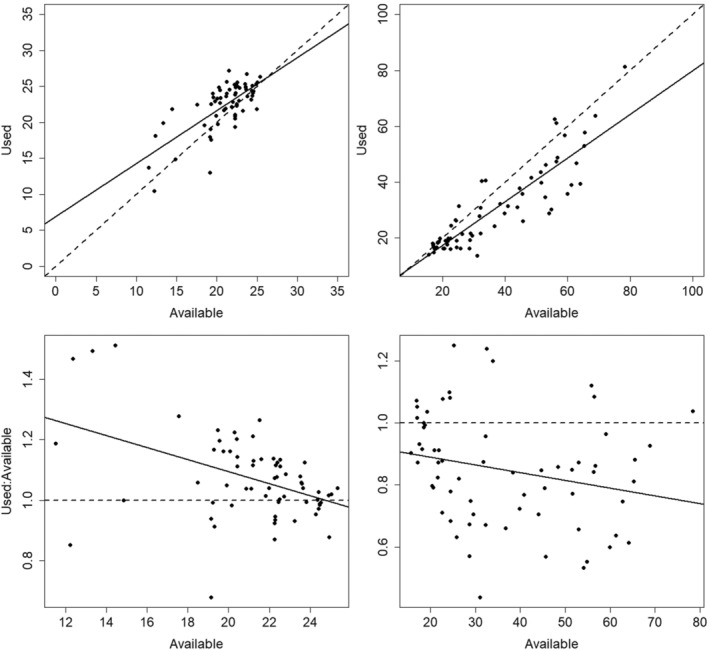
Additional univariate functional response relationships observed from greater sage‐grouse late brood selection analyses for eastern Idaho, USA, during 2015–2020. Shown are results from additive scale habitat use analyses (approach 1) regressing average used conditions against average available conditions (top row) for the following variables: litter cover (300 m scale; left), and percent cover of bare ground (300 m scale; right). Also shown are the multiplicative scale habitat use relationships (bottom row), with used: available habitat (i.e., selection ratios) regressed against average available conditions for each variable. Dashed lines represent a 1:1 relationship between used and available habitat for each variable and hence proportional use. Ecological interpretations of functional responses are provided in Table 4.

**FIGURE A5:**
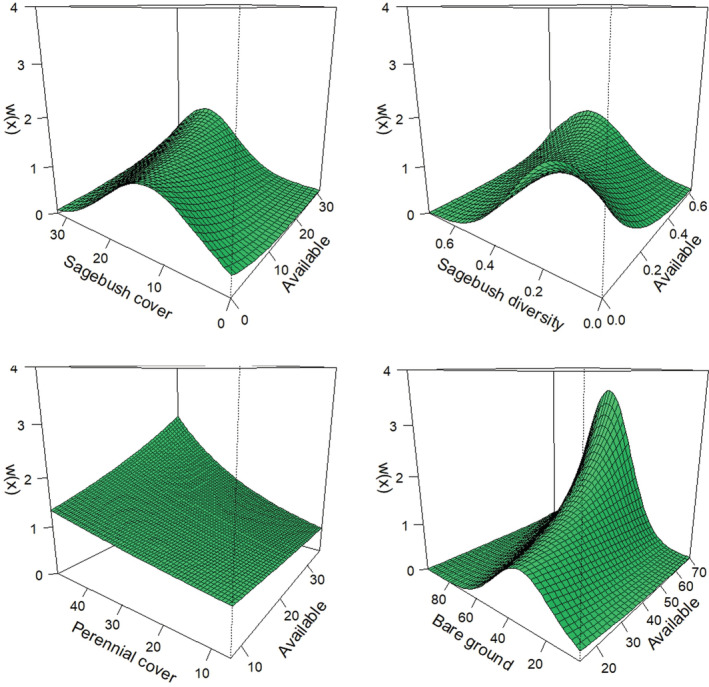
Additional functional response interactions observed from early brood‐rearing resource selection for greater sage‐grouse in eastern Idaho, USA, during 2015–2020. Results are interactions from the optimal multiscale resource selection function (analysis approach 4) for percent sagebrush cover (1000 m scale; top left), diversity of sagebrush cover categories (800 m scale; top right), percent cover of perennial herbs (200 m scale; bottom left), and percent cover of bare ground (100 m scale; bottom right), where the response variable w represents the relative probability of use. Interpretations of functional response interactions are described in Table 4.

**FIGURE A6:**
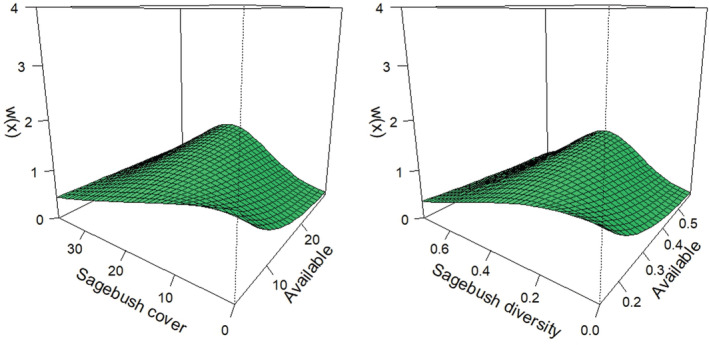
Additional functional response interactions observed from late brood‐rearing resource selection for greater sage‐grouse in eastern Idaho, USA, during 2015–2020. Results are interactions from the optimal resource selection function (analysis approach 4) for percent sagebrush cover (100 m scale; left) and diversity of sagebrush cover categories (700 m scale; right), where the response variable w represents the relative probability of use. Interpretations of functional response interactions are described in the text and in Table 4.

The Grassy Ridge Fire produced strong changes in habitat availability for many vegetation variables, and functional responses in the use and selection of resources by breeding sage‐grouse were common (Tables 3 and 4; Figures 5, 6, 7, A1, A2, A3, A4, A5, A6). Univariate analyses demonstrated that functional responses in habitat use were common for nesting females, where a variety of responses were observed for both remotely sensed and locally measured habitat variables (Table 3; Figures 5 and 6, A1 and A2). Tradeoffs, where use was greater than availability at low values of availability but less than availability as availability of the resource increased, were observed for the heterogeneity in sagebrush cover classes and annual herbaceous cover measured using remote sensing, and for perennial grass height measured locally surrounding nests. Relaxed responses, where resource use was either greater (selection) or less than (avoidance) availability when average resource availability was low, but approached proportional use as availability increased, was observed for several variables, including sagebrush cover and height measured using remote sensing, and sagebrush cover, shrub cover, perennial forb height, and forb cover measured locally at nests. For example, the average sagebrush cover measured at nests remained at >25% even when the average available cover was <15%, but the average used cover approached availability as the average available cover increased to >25% (Figure 5). Similarly, we did not observe any nests inside the fire perimeter after the burn, where the average sagebrush cover measured from remotely sensed data dropped from 24% preburn to <1% postburn. Moreover, results were generally consistent for additive (approach 1) and multiplicative (approach 2) measures of habitat use, with the exception of the remotely sensed cover of bare ground (decreased use, proportional selection) and forb richness measured locally surrounding nests (increased use, proportional selection). Similarly, results of additive and multiplicative scale tests for functional responses were mostly consistent across spatial scales (Tables 3 and 4; scale sensitivity results are provided in Stevens et al., 2023).

**TABLE 3:** Types of functional responses observed for habitat use by nesting greater sage‐grouse, in eastern Idaho, USA, during 2015–2020.

Variable	Additive use (approach 1)			Multiplicative use (approach 2)			RSF model with interaction (approach 4)	% change
	$\beta_{0}$	$\beta_{1}$	Interpretation	$\beta_{0}$	$\beta_{1}$	Interpretation	Interpretation	
Remotely sensed data								
Sagebrush cover	>0	1, <1^a^	Proportional, Relaxed	>0	<1	Relaxed	Relaxed^b^	−42
Shrub cover	≠0^a^	1	Additive	0, ≠0^c^	1	Proportional, additive	NA^d^	−42
Diversity of sagebrush cover	>0^e^	<1^e^	Tradeoff	<0^e^	<1^e^	Tradeoff	Tradeoff, Decreased^f^	−1
Sagebrush height	>0	<1	Relaxed	>0	<1	Relaxed	NA	−31
Herbaceous cover	0	1	Proportional	0, ≠0^g^	1	Proportional, additive	NA	−15
Perennial herb cover	0	1	Proportional	0, ≠0^g^	1	Proportional, additive	Tradeoff	−15
Annual herb cover	>0^a^	<1^e^	Tradeoff	0	1, <1^h^	Proportional, tradeoff	Additive	+5
Litter cover	>0	<1	Relaxed	>0	<1	Relaxed	Relaxed	−8
Bare ground	0	<1	Decreased	0	1	Proportional	Tradeoff	+84
Transect data								
Sagebrush cover	>0	<1	Relaxed	>0	<1	Relaxed	NA	−32
Shrub cover	>0	<1	Relaxed	>0	<1	Relaxed	NA	−35
Sagebrush height	≠0	1	Additive	≠0	1	Additive	NA	−31
Perennial grass cover	0	1	Proportional	0	1	Proportional	Additive	−28
Perennial grass height	>0	<1	Tradeoff	>0	<1	Tradeoff	Relaxed, decreased^i^	−12
Perennial forb height	>0	<1	Relaxed	>0	<1	Relaxed	Additive	+50
Forb cover	>0	<1	Relaxed	>0	<1	Relaxed	Proportional	+5
Forb richness	0	>1	Increased	0	1	Proportional	Additive	0
Annual grass cover	>0	<1	Tradeoff	0	1	Proportional	NA	+10

*Note*: Results are provided for each remotely sensed and locally measured (Transect) vegetation attribute (Variable). Results for each variable are provided for each statistical method described in the text. Functional response types observed (Interpretation) were deduced from linear regression parameter values ($\beta_{0}$, $\beta_{1}$) and graphical relationships observed (Tables 1 and 2, Figure 4) for analysis approaches 1 (additive scale) and 2 (multiplicative scale), and from graphical predictions of the relative probability of selection generated from interaction terms of resource selection models (analysis approach 4). No functional response was indicated by either proportional (i.e., resource used in proportion to available, no selection) or additive (i.e., selection or avoidance that did not change with availability) habitat use. Also included are the percent changes (% change) in average resource values measured from the availability samples (at random points) prefire and postfire on the Sand Creek study area.

^a^ Graphical relationships and interpretation consistent across scales, statistical differences (i.e., from 0 or 1) for 7 of 10 scales. For sagebrush cover, the confidence interval boundary was 1.0 for 7 of 10 scales, with the same pattern but less precision for the remaining 3 scales.

^b^ Relaxed selection of high‐cover patches (100 m scale) as average available cover increases, relaxed avoidance of sparse cover patches as average available cover increases.

^c^ Statistically different from 0 at 5 largest scales (additive use) but not different from 0 at 5 smallest scales (proportional use).

^d^ NA represents a variable not included in the final resource selection function.

^e^ Statistically different for 9 of 10 scales (all scales > 100 m).

^f^ Tradeoff responses for patches (1000 m scale) of homogeneous sagebrush cover (selection when homogeneous patches were rare, avoidance when abundant), decreased selection for patches of heterogeneous sagebrush cover (stronger avoidance when heterogenous patches were more abundant).

^g^ Statistically different from 0 for only the largest scale (1000 m scale; additive use), no different from 0 otherwise (proportional use).

^h^ Statistically less than 1 for 5 scales (tradeoff), 1 otherwise (proportional use).

^i^ Relaxed selection of shorter grass as average available grass height decreased (i.e., selection approached proportional use), decreased selection of taller grass as average available grass height increased.

**TABLE 4:** Types of functional responses observed for habitat use by brood‐rearing greater sage‐grouse, in eastern Idaho, USA, during 2015–2020.

Variable	Additive use (approach 1)			Multiplicative use (approach 2)			RSF model with interaction (approach 4)	% change
	$\beta_{0}$	$\beta_{1}$	Interpretation	$\beta_{0}$	$\beta_{1}$	Interpretation	Interpretation	
Early brood rearing								
Sagebrush cover	0, ≠0^a^	1	Proportional, additive	0	1	Proportional	Relaxed^b^	−5
Shrub cover	0, ≠0^c^	1	Proportional, additive	0	1	Proportional	NA	−4
Diversity of sagebrush cover	0	1	Proportional	0	1	Proportional	Complex^d^	+11
Sagebrush height	0	1	Proportional	<0^e^, 0	>1, 1	Relaxed, proportional	NA	−1
Herbaceous cover	0	1	Proportional	0	1	Proportional	NA	−1
Perennial herb cover	0	1	Proportional	0	1	Proportional	Increased, relaxed^f^	0
Annual herb cover	<0	>1	Relaxed^g^	<0	>1	Relaxed	Relaxed	−4
Litter cover	0	1	Proportional	0	1	Proportional	Additive	−11
Bare ground	0	<1^h^	Decreased	0	1	Proportional	Complex^i^	+10
Late brood rearing								
Sagebrush cover	≠0	1	Additive	0	1	Proportional	Complex^j^	−11
Shrub cover	≠0	1	Additive	0	1	Proportional	NA	−8
Diversity of sagebrush cover	0	1	Proportional	0	1	Proportional	Complex^k^	+1
Sagebrush height	≠0	1	Additive	0	1	Proportional	NA	−10
Herbaceous cover	0	1	Proportional	0	1	Proportional	NA	+1
Perennial herb cover	0	1	Proportional	0	1	Proportional	Additive	+2
Annual herb cover	0	1	Proportional	≠0	1	Additive	Additive	−16
Litter cover	>0	<1	Relaxed	0	<1	Relaxed^l^	Additive	−4
Bare ground	0	<1	Decreased	0	<1	Decreased	Complex^m^	+12

*Note*: Results are provided for each remotely sensed vegetation attribute (Variable), and results for each variable are provided for each statistical method described in the text. Functional response types observed (Interpretation) were deduced from linear regression parameter values ($\beta_{0}$, $\beta_{1}$) and graphical relationships observed (Tables 1 and 2, Figure 4) for analysis approaches 1 (additive scale) and 2 (multiplicative scale), and from graphical predictions of the relative probability of selection generated from interaction terms of resource selection functions (RSF; analysis approach 4). No functional response was indicated by either proportional (i.e., resource used in proportion to available, no selection) or additive (i.e., selection or avoidance that did not change with availability) habitat use. The functional response interaction was labeled complex (and described in footnotes) for RSF interaction surfaces that did not clearly fit into existing functional response categories. Also included are the percent changes (% change) in average resource values measured from the availability samples (at random points) prefire and postfire on the Sand Creek study area.

^a^ Additive use found for the three largest spatial scales, proportional use otherwise.

^b^ Response surface as a whole is conceptually similar to relaxed selection, with selection for intermediate amounts of cover at low availability and selection for cover approximately equal to average conditions at high availability.

^c^ Additive use found for one spatial scale (800 m), proportional use otherwise.

^d^ Surface shows selection for patches with more homogeneous sagebrush cover classes when areas around nests were more homogeneous (avoidance otherwise), and selection for patches with more diversity of sagebrush cover classes when areas around nests are more diverse (avoidance otherwise).

^e^ Relaxed avoidance for 5 scales (200–600 m), proportional use otherwise.

^f^ Relaxed avoidance of low‐cover patches (avoidance when rare, proportional use when common) but increased use of high‐cover patches (positive selection that gets stronger with availability).

^g^ Statistically different for 9 of 10 scales, with the same pattern of relaxed avoidance but less precision for the remaining scale.

^h^ Statistically significant for 7 of 10 scales, with the same pattern but less precision otherwise.

^i^ Surface shows selection for intermediate amounts of bare ground at fine scales (100 m) when the average amount of bare ground near nests is low, but as the average amount of bare ground increases, there was increased intensity of selection for patches with bare ground coverage similar to average conditions.

^j^ Surface shows selection for low‐cover patches at fine scales (100 m) when average cover is low but strong avoidance of these patches as availability increased, and selection for cover approximately equal to average conditions at high availability.

^k^ Surface shows selection for low diversity patches when average diversity of cover classes was low but strong avoidance of these patches as average diversity increased, and selection for the diversity of sagebrush cover classes approximately equal to average conditions as average diversity increased.

^l^ Graphical results imply relaxed selection, and the regression slope is <1 for all scales. The intercept terms were >0, but statistically are no different than 0.

^m^ Surface shows selection for intermediate amounts of bare ground (300 m scale) when the average amount of bare ground was low in the landscape surrounding nests, yet there was a strong tradeoff response for patches with small amounts of bare ground, which were avoided when common but strongly selected when rare (i.e., when average cover of bare ground increased).

Univariate analyses revealed functional responses by sage‐grouse during brood rearing but also suggested functional responses may be less common at this stage than for nesting (Table 4; Figure 7). After conditioning on nest locations, changes in available resources for early and late brood rearing were also less pronounced postfire (Table 4). Specific responses differed between early and late brood rearing, yet observed functional responses were again usually consistent between the additive (approach 1) and multiplicative (approach 2) measures of habitat use. During early brood rearing, we observed relaxed avoidance of annual herbaceous cover and decreased use of bare ground (additive scale only). Relaxed avoidance of tall sagebrush was also observed during early brood rearing but only over a specific range of spatial extents (200–600 m). During late brood rearing, we observed a relaxed selection of litter cover, and decreased use of bare ground (both additive and multiplicative scales of use).

### 3.2 Predicting resource selection

**FIGURE A7:**
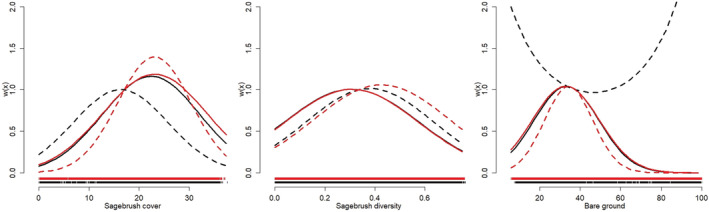
Comparison of fitted selection relationships from the final resource selection function (RSF) for greater sage‐grouse during late brood rearing (solid lines), projected to the mean available conditions on the Sand Creek study area of eastern Idaho, USA, prior to (black) and after (red) the Grassy Ridge Fire. Also shown are fitted relationships from the same RSF fit using only prefire data (dashed lines) but projected to the mean postfire available conditions on the Sand Creek study area, both including (red) and excluding (black) the functional response interaction terms. Results are presented for covariates where functional responses were supported in brood‐rearing RSF analyses (see Table 4): percent sagebrush cover (100 m scale; left), diversity of sagebrush cover classes (700 m scale; middle), and percent cover of bare ground (300 m scale; right). Black and red vertical tick marks along the *x*‐axis represent observed covariate values at available locations for the entire data set (red) and the prefire (black) observations.

Controlling for partial effects of vegetation covariates facilitated additional insight into functional responses through predictive RSF modeling (Tables 3 and 4; Figures 5, 6, 7, A5, A6, A7). Inferences from GFR models were often consistent with functional response results from univariate analyses during the nesting period (e.g., sagebrush cover, litter cover) but also revealed more complex and subtly different relationships. Fitted functional response interaction surfaces from predictive models generally provided a more complicated interpretation, as the interpretation of 2‐dimensional portrayals of the 3‐dimensional interaction surfaces could change with the location in the parameter space (i.e., where along the covariate axis the selection changes with availability are assessed; Table 3, Figure 6). Approach 4 also suggested functional responses for nesting and brood‐rearing habitat where univariate analyses failed to detect relationships (e.g., perennial herbaceous cover during nesting; Tables 3 and 4). This was especially true for brood rearing, where RSF models included functional responses for sagebrush cover, diversity of sagebrush cover, and perennial herbaceous cover, whereas univariate analyses indicated no functional responses for these variables. Optimal spatial extents also varied among vegetation covariates from the smallest (100 m) to largest (1000 m) extents considered (Tables A1, A2, A3), and final RSFs were multiscale and included each variable at its optimal scale (supporting RSF results are provided in Stevens et al., 2023). Final RSFs also included control variables (Tables A1, A2, A3), where the estimated effects were typically as expected (e.g., negative effects for distance to lek and distance to mesic grass patch on nest‐site selection, negative effects for roughness and distance to mesic grass patch on late brood selection) or biologically insignificant. Final RSF models resulted in out‐of‐sample average test AUC scores (across cross‐validation folds) of 0.73, 0.63, and 0.69 for nest site, early brood, and late brood selection, respectively. Sensitivity analyses showed that data thinning to a maximum of three locations per day per individual had minimal effect on RSFs, where coefficient estimates were little changed from RSFs generated using the entire data set (Tables A2, A3).

**FIGURE 8:**
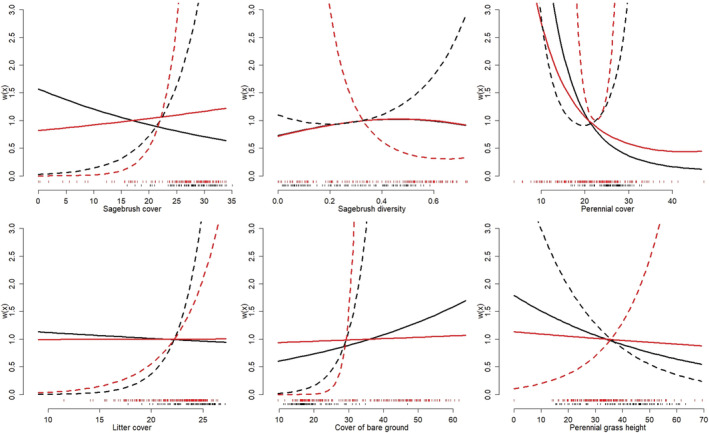
Comparison of fitted selection relationships from the final resource selection function (RSF) for nesting greater sage‐grouse (solid lines) projected to the mean available conditions on the Sand Creek study area of eastern Idaho, USA, prior to (black) and after (red) the Grassy Ridge Fire. Also shown are fitted selection relationships from the same RSF fit using only prefire data (dashed lines) but projected to the mean postfire available conditions on the Sand Creek study area, both including (red) and excluding (black) the functional response interaction terms. Results are presented for the covariates where functional responses were supported in RSF analyses: percent sagebrush cover (100 m scale; top left), diversity of sagebrush cover classes (1000 m scale; top middle), percent cover of perennial herbs (500 m scale; top right), percent litter cover (1000 m scale; bottom left), percent cover of bare ground (1000 m scale; bottom middle), and perennial grass height (transect scale; bottom right). Black and red vertical tick marks along the *x*‐axis represent observed covariate values at available locations for the entire data set (red) and the prefire observations (black).

**FIGURE 9:**
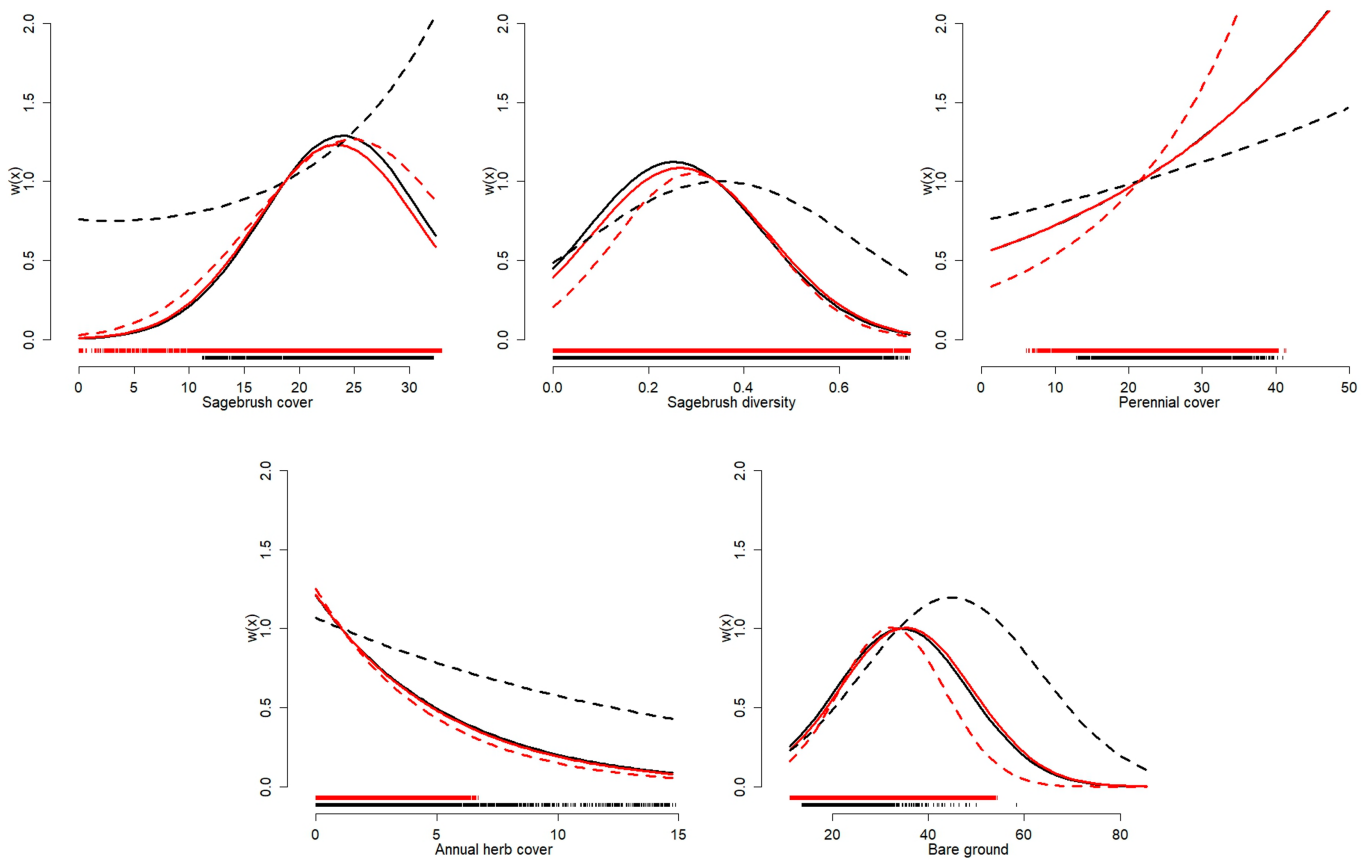
Comparison of fitted selection relationships from the final resource selection function (RSF) for greater sage‐grouse during early brood rearing (solid lines), projected to the mean available conditions on the Sand Creek study area of eastern Idaho, USA, prior to (black) and after (red) the Grassy Ridge Fire. Also shown are fitted selection relationships from the same RSF fit using only prefire data (dashed lines) but projected to the mean postfire available conditions on the Sand Creek study area, both including (red) and excluding (black) the functional response interaction terms. Results are presented for the covariates where functional responses were supported in RSF analyses: percent cover of sagebrush cover (1000 m scale; top left), diversity of sagebrush cover classes (800 m scale; top middle), percent cover of perennial herbs (200 m scale; top right), percent cover of annual herbs (500 m scale; bottom left), and percent cover of bare ground (100 m scale; bottom right). Black and red vertical tick marks along the *x*‐axis represent observed covariate values at available locations for the entire data set (red) and the prefire (black) observations.

Comparison of selection relationships from the final RSF with those fit using only data collected before the Grassy Ridge Fire showed that nest‐site selection relationships estimated using only prefire data typically differed strongly from the final model (i.e., using all data and where covariate values covered a broader range of conditions; Figure 8). Such differences were common even when models fit to prefire data also included the functional response interaction terms. Fire‐induced changes in average available habitat shaped functional responses for several variables (sagebrush cover, bare ground, perennial grass height), whereas functional responses for other variables appeared less directly affected by changes in average availability before and after the fire (e.g., diversity of sagebrush cover; Figure 8). By contrast, differences in selection relationships between models fit with prefire data and the entire data set were less pronounced for brood selection (Figures 9 and A7). The observed functional responses for brood selection also appeared to be driven more by individual variation in average available conditions (i.e., due to their individual nest locations), rather than by changes in average available conditions resulting directly from the fire (Figures 9 and A7).

**FIGURE 10:**
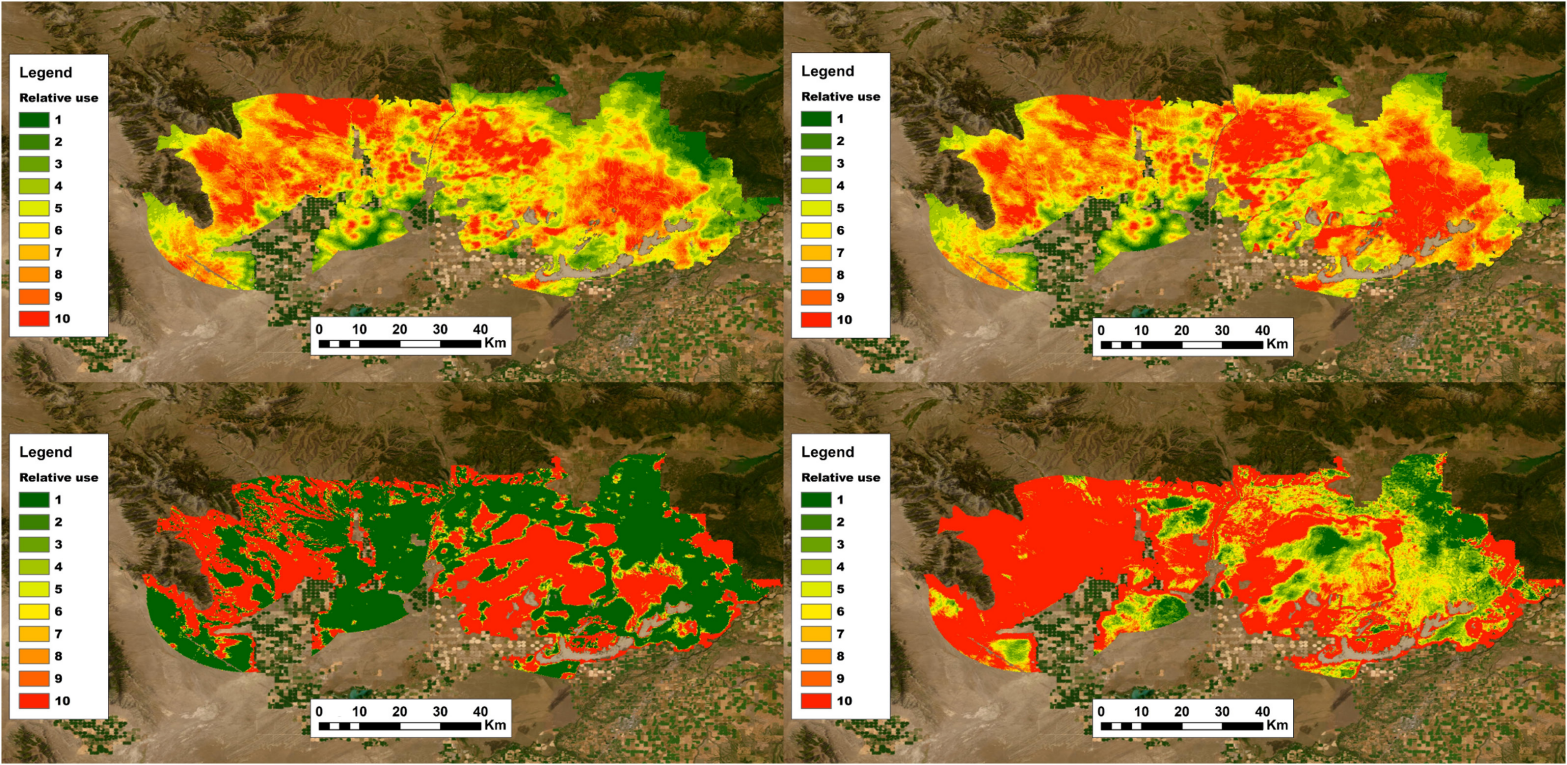
Mapped predictions from optimally‐predictive resource selection function (top row) for nesting greater sage‐grouse in eastern Idaho, USA, before (left) and after (right) the Grassy Ridge Fire. Relative probability of use is mapped in ordinal categories from low (1) to high (10), incorporating observed functional responses and with category boundaries set using prefire data to express the change in the relative probability of selection on the same scale and relative to prefire conditions (see Predicting Resource Selection). Also shown are mapped predictions from resource selection functions fit using only prefire data but mapped onto postfire conditions (bottom row), both with (left) and without (right) inclusion of functional response interaction terms in the model.

**FIGURE A8:**
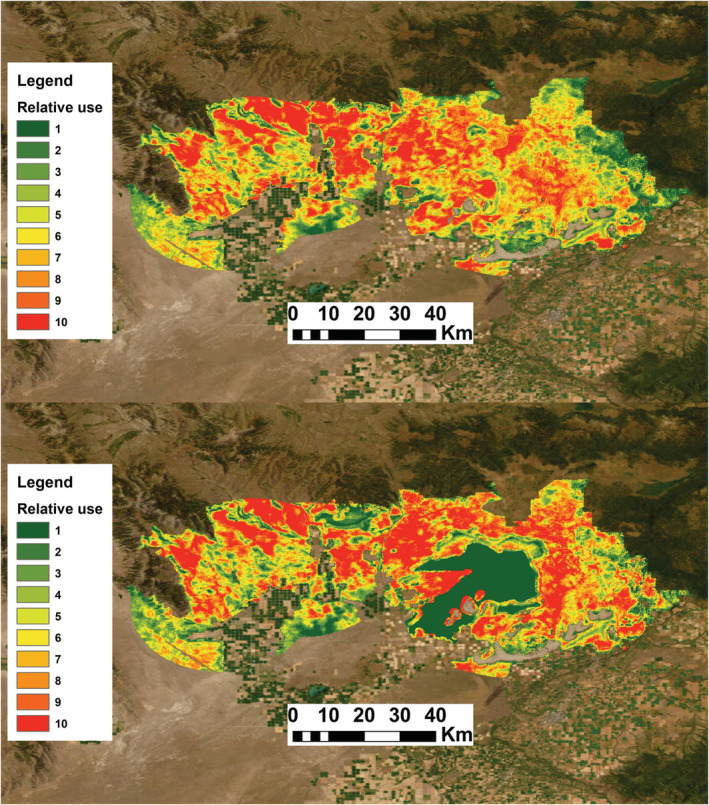
Composite resource selection function (RSF) for breeding greater sage‐grouse in eastern Idaho, USA, before (top) and after (bottom) the Grassy Ridge Fire. Relative probability of use is mapped in ordinal categories from low (1) to high (10), incorporating observed functional responses for all breeding season RSFs by multiplying individual RSFs (i.e., for nesting, early brood rearing, and late brood rearing), effectively weighting brood‐rearing use predictions based on probability of nesting at a given location (See Appendix S1). Category boundaries were set using prefire data to express the change in the relative probability of selection on the same scale and relative to prefire conditions.

**FIGURE A9:**
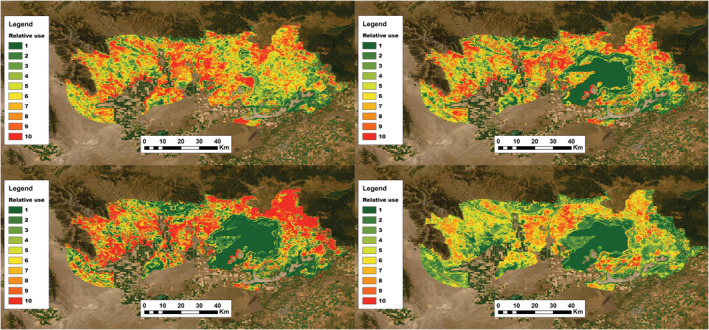
Mapped predictions from optimally‐predictive resource selection function for greater sage‐grouse during early brood‐rearing (top row), before (left), and after (right) the Grassy Ridge fire. Also shown are mapped predictions from resource selection functions fit using only prefire data (but mapped onto postfire conditions; bottom row), both with (left) and without (right) inclusion of functional response interaction terms in the model.

**FIGURE A10:**
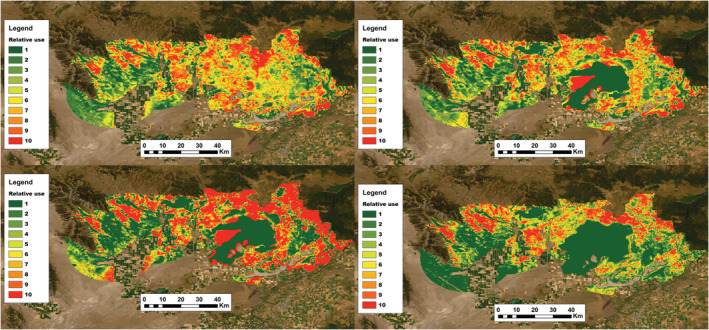
Mapped predictions from optimally‐predictive resource selection function for greater sage‐grouse during late brood‐rearing (top row), before (left), and after (right) the Grassy Ridge fire. Also shown are mapped predictions from resource selection functions fit using only prefire data (but mapped onto postfire conditions; bottom row), both with (left) and without (right) inclusion of functional response interaction terms in the model.

Nesting RSF maps showed that the Grassy Ridge Fire had strong negative impacts on predicted space use within and around the burn perimeter, but the fire also increased the strength of selection for the remaining intact sagebrush on the northern and eastern portions of Sand Creek (Figure 10). Specifically, the burn reduced the intensity of selection for nesting habitat inside the fire perimeter and increased the intensity of selection for remaining portions of Sand Creek. Similar patterns were observed for early and late brood‐rearing periods (Figures A8, A9, A10). Mapped RSF predictions from models built using only prefire data also resulted in very different predictions of space use as compared to the entire data set (Figures 10, A9, A10). Lastly, the fit of individual RSF maps to the observed use data varied among the three stages of the breeding period but was better for nesting than brood rearing (nesting: ρ = .98 (prefire), ρ = .96 (postfire); early brood: ρ = .61 (prefire), ρ = .81 (postfire); late brood: ρ = .61 (prefire), ρ = .41 (postfire)), and was considerably worse when RSF models generated using only prefire data were used to predict postfire use (nesting: ρ = −.36 (including functional responses), ρ = .19 (excluding functional responses); early brood: ρ = .62 (including functional responses), ρ = .01 (excluding functional responses); late brood: ρ = −.03 (including functional responses), ρ = −.54 (excluding functional responses)). Moreover, the composite RSF that aggregated final model predictions across nesting and brood‐rearing periods (Figure A8) provided a good fit to all use locations (prefire: ρ = .96; postfire: ρ = .94).

## 4 DISCUSSION

This work demonstrated that high‐severity megafire can have strong effects on available resources and subsequent patterns of space use, by eliminating large swaths of nesting habitat but also by modifying patterns of use and selection for remaining habitat. We observed a variety of functional responses across different vegetation components, at multiple spatial scales and for multiple stages of the breeding season, including use and selection of nest sites and early and late brood‐rearing habitat. Vegetation available for nesting was strongly affected by megafire, and functional responses observed for the use and selection of nesting resources were influenced by landscape‐level changes in availability. After conditioning on the selection of nest sites, functional responses in brood habitat use appeared to be driven by spatial–temporal variation in available resources among individual females. We also incorporated functional responses into RSFs to map the pixel‐scale intensity of selection prefire and postfire, both to understand the temporal change in the distribution of habitat and also to aid in habitat management and restoration efforts. The relative probability of selection predicted for each of the three RSFs (nesting, early brood, and late brood) uniquely accounted for changing environmental conditions and average available habitat measured at a variety of spatial scales, where we optimized the extent of each covariate, along with overall model complexity, for out‐of‐sample prediction. Importantly, habitat relationships and their mapped predictions from RSFs lacked transferability to a post‐megafire landscape when built using only data collected before the Grassy Ridge Fire. Consequently, a more complete understanding and better prediction of space use in the aftermath of megafire was facilitated through the lens of functional responses, as space use patterns were not static in the face of large‐scale environmental change.

Our results show that functional responses are a useful framework for understanding the effects of disturbance on patterns of habitat use in a rapidly changing world. Functional responses by breeding sage‐grouse were common as resource availability changed, demonstrating that the context of changing habitat via large‐scale disturbance can affect patterns of use and selection of remaining vegetation. Other researchers demonstrated functional responses in animal space use as a consequence of human disturbance, primarily for large mammals. For example, functional responses proved useful in describing the selection of forested habitats in response to disturbance by logging for woodland caribou (*Rangifer tarandus caribou*; Moreau et al., 2012) and Canada lynx (*Lynx canadensis*; Holbrook et al., 2017). Functional responses also shaped habitat use by cougars (*Puma conclor*; Knopff et al., 2014), wolves (*Canis lupus*), and caribou (Mumma et al., 2019) in response to anthropogenic infrastructure, and the responses of wolverines (*Gulo gulo*; Heinemeyer et al., 2019) to motorized recreation. We believe our study is the first to document functional responses to vegetation changes induced by a high‐severity megafire, which is a consequence of global change with strong implications for biodiversity conservation (Abatzoglou & Williams, 2016; Duane et al., 2021; McKenzie et al., 2003). We also demonstrated the use of multiple, complimentary analyses of functional responses for habitat use and selection, an approach that is generalizable across taxa and facilitates a deeper understanding of space use in the presence of changing environmental conditions (Holbrook et al., 2017, 2019).

This study also demonstrates the difficulty of predicting space use responses to future disturbance by showing that context‐dependent habitat use varies by the resource in question, and that predisturbance habitat models should be viewed cautiously when used to extrapolate to postdisturbance landscapes for management planning purposes. We documented a variety of functional responses (e.g., relaxed, tradeoff, increased and decreased use or selection) that changed among the specific components of vegetation and between nesting and brood rearing. This heterogeneity of responses to individual resources, along with the commonality where functional responses were consistently observed across resources, complicates the transferability of habitat models to new locations in space and time, especially in the presence of changing conditions. Indeed, predictive RSFs built using only prefire data had very poor transferability to post‐megafire conditions, resulting in unstable selection relationships and unreliable mapped predictions. This lack of transferability was apparent irrespective of whether or not the models contained functional response interaction terms. Thus, statistically controlling for changes in availability through functional response interactions may not be enough, and observing space use over a broader range of environmental conditions than exist prior to disturbance may also be necessary for making reliable predictions in a rapidly changing environment (Avgar et al., 2020; Holbrook et al., 2019; Matthiopoulos et al., 2011). As such, observing a broad contrast of available environmental conditions, either before and after disturbance or by incorporating additional study sites (or both) may be necessary to improve RSF predictions and the management planning tools deduced from them. Similarly, while mapped predictions from models built using the entire data set provided a reasonable fit to the observed use locations from this study, our results imply that extrapolating RSF predictions from models developed under different levels of disturbance may not produce reliable predictions in novel landscapes generated by disturbance events.

Wildfire has increased in frequency, severity, and scale in recent decades across the sagebrush biome (Baker, 2006; Balch et al., 2013; Pilliod et al., 2017). Despite known detrimental effects of wildfire on sagebrush‐steppe plant and animal communities (Holbrook et al., 2016; Knick et al., 2003; Reeves et al., 2018), the ways in which large‐scale wildfire modifies patterns of animal space use in the vegetation patches that remain are less understood. Sage‐grouse are commonly thought of as an indicator species for the conservation of endemic sagebrush wildlife (Rowland et al., 2006), and disturbances affecting the habitat for sage‐grouse also affect sympatric species. For example, watershed‐level removal of trees in areas with conifer encroachment has been implemented to improve the habitat for sage‐grouse (Severson et al., 2017) but also affected habitat quality for sympatric songbirds (Donnelly et al., 2017; Holmes et al., 2017). The commonality of functional responses for sage‐grouse therefore suggests that changes in habitat availability induced by large‐scale disturbance could influence the use and selection of remaining habitat patches by other endemic wildlife. If correct, then characterizing functional responses for a broader suite of sagebrush wildlife could facilitate better understanding and prediction of responses of this community to wildfire (Aarts et al., 2013; Holbrook et al., 2019).

Many functional responses we observed for breeding sage‐grouse have intuitive and straightforward interpretations. For example, sage‐grouse exhibited relaxed use and selection of several nesting resources, with disproportionate use of resources when they were scarce on the landscape that relaxed and approximated proportional use as they became more available. This was observed for both fine‐scale variables measured directly around nests (sagebrush cover, shrub cover, forb height, and forb cover) and remotely sensed resources measured across a variety of spatial extents (sagebrush cover and height, litter cover). Relaxed selection is expected for specialist species that require specific amounts or types of habitat because they will seek that habitat out even if it is not widely available (Holbrook et al., 2019). Thus, the relaxed selection of shrubs for nesting by a sagebrush‐obligate species like sage‐grouse was anticipated. For example, average sagebrush cover at the nest site remained >25%, even when fire reduced average available cover across the study area and effectively eliminated sagebrush cover inside the fire perimeter. Tradeoff responses were also observed for multiple resources, including perennial grass height measured directly around nests and diversity of sagebrush cover classes and annual herbaceous cover measured from remote sensing. Tradeoff responses showed disproportionately high use of resources that were scarce but switched to disproportionately low use as they became more available. Therefore, taller grasses were used at fine scales when grass height was shorter on average, but the opposite was true as average height increased, likely due to a tradeoff with other important nesting resources (e.g., sagebrush cover). Similarly, when the diversity of sagebrush cover classes was reduced across the burned area (i.e., after the burn, which effectively reduced the fire scar to one class), use was disproportionately high in patches outside the burn with more diversity of sagebrush cover. In addition to relaxed and tradeoff responses, sage‐grouse also exhibited decreased use of bare ground during both nesting and brood rearing, whereas the cover of bare ground increased across the study area after the burn.

Although many observed functional responses had consistent interpretations across analyses, this was not always the case and some relationships differed depending on the metric or method used, or the spatial scale of observation. For example, additive scale analyses (approach 1) of nesting habitat provided evidence for decreased use of areas with bare ground as it became more available on the landscape after the burn, but multiplicative scale analyses (approach 2) failed to provide statistical evidence for disproportionate selection. Similarly, additive scale analyses provided evidence for increased use of fine‐scale forb richness as average forb richness increased, whereas multiplicative scale analyses again failed to provide evidence for disproportionate selection. Differences in results for additive and multiplicative scale analyses under increased and decreased use scenarios are common (Holbrook et al., 2019) because additive use is measured and can change distinctly from availability, whereas selection ratios are a mathematical function of available conditions (Lele et al., 2013). Consequently, functional responses resulting from increased or decreased use cannot be inferred on the multiplicative scale.

Different types of functional responses are not inferred using only statistical results, but rather involve formal statistical tests in tandem with graphical interpretation because regression coefficients produced by different response types are not mutually exclusive (Tables 1 and 2; Figure 4). This means that some functional response types can be challenging to differentiate with real data, and interpretation depends on the observed contrast in average available conditions (e.g., differentiating tradeoff vs. relaxed responses). Thus, classification of functional response types involves some inherent subjectivity, which has been implicitly acknowledged by others (Holbrook et al., 2019). The functional response relationships we observed were also mostly consistent across spatial scales, but responses to changing availability were inconsistent for some variables (e.g., annual herb cover). Moreover, while we assessed functional responses over a relevant range of spatial scales for each variable, the optimal scale for multiple variables was the largest spatial extent we considered. This suggests that measuring some variables at even larger extents (>1000 m) could have resulted in better prediction, or possibly different inferences (Jackson & Fahrig, 2015). Therefore, spatial scales of observation should be carefully considered relative to study goals and species life history when assessing the impacts of disturbance on patterns of use and selection, and evaluation of broader spatial scales than we considered here appears warranted.

Our use of RSFs was primarily intended for predictive modeling, and the interactions between used and available resources fit using generalized functional response models often provided the most complicated ecological interpretation. The interactions produce three‐dimensional response surfaces predicting the relative probability of selection as a function of both the resource value at a pixel and average available conditions, and our results imply the ecological interpretation of functional response types derived from these plots can depend on where one chooses to take a two‐dimensional slice through the three‐dimensional surface (i.e., across the plane of average available conditions). This issue is often not addressed by researchers characterizing types of functional responses observed within RSFs, and it is often unclear where exactly on the three‐dimensional response surface the two‐dimensional graphical representations are arising from (e.g., figures 2 and 3 of Holbrook et al., 2019). This suggests that the predefined and ecologically intuitive categories of functional responses (e.g., relaxed, tradeoff) may at times be over‐simplifications of complex and nonlinear changes to the predicted intensity of habitat selection, and that functional responses may not cleanly fit into existing categories described in the literature. Regardless of their complexity, the RSF response surfaces clearly showed potential for strong changes to predicted space use that depend on the context of available resources that are changing across landscapes with environmental conditions and disturbance events.

Collectively, our results provide direct evidence for the context‐dependent use of resources by sage‐grouse, where the use of nesting habitat is shaped by the available environmental conditions, which corroborates the results of recent studies (O'Neil et al., 2021; Schuyler et al., 2022; Smith et al., 2020). For example, a meta‐analysis of nest‐selection studies implemented across the sage‐grouse range found evidence for functional responses in selection for sagebrush cover, total shrub cover, shrub height, and live grass height (Smith et al., 2020). O'Neil et al. (2021) reported changes in nest‐site selection by sage‐grouse across the Great Basin as a function of changes to an index of ecosystem resistance and resilience (a function of soil temperature and moisture). This analysis allowed for regional heterogeneity in selection coefficients among populations but did not assess changes in selection as a function of changes to the availability of individual resources (O'Neil et al., 2021). Despite differences in methods, scales of observation, and level of organization (i.e., within vs. among populations), our results corroborate these findings and provide further evidence for context‐dependent habitat use for a variety of vegetation resources during the breeding season. We also demonstrate that context‐dependent use can be induced within a single population in response to abrupt changes in available resources caused by large‐scale disturbance events (see also Schuyler et al., 2022). Furthermore, the context dependence we observed implies that other disturbance events that change the composition and structure of available vegetation in sagebrush ecosystems (e.g., mechanical brush treatment) could result in functional changes in space use by sage‐grouse and other wildlife.

Context‐dependent habitat use also suggests some plasticity in breeding season space use by sage‐grouse. We observed functional responses in the use of multiple resources, which demonstrates that use and selection patterns are not static. While other studies demonstrated functional responses in nest‐site selection, primarily among different sage‐grouse populations that were exposed to unique landscapes (O'Neil et al., 2021; Smith et al., 2020), our results demonstrate that context‐dependent space use can also be observed within a population when it is exposed to changing conditions over time. We also observed multiple instances of context‐dependent habitat use on the additive scale, demonstrating that functional responses were not merely an artifact of changing availability. This was true for use of multiple components of nesting habitat (e.g., sagebrush cover and height, bare ground) but was also observed to a lesser extent during brood rearing (e.g., annual herbs, bare ground). Temporal changes in space use after a megafire, but without prefire data for comparison, were also recently observed across multiple seasonal periods for sage‐grouse in Oregon (Schuyler et al., 2022). Regardless of whether responses resulted from temporal changes in behavior or simply as artifacts of changing resource availability over time, our results demonstrate benefits of functional response analyses to facilitate a better understanding of space use in changing environments.

While our results demonstrate rapid changes in habitat use by breeding sage‐grouse, this does not mean that all individuals will necessarily respond to changing conditions immediately. For example, Foster et al. (2019) reported that sage‐grouse females typically nested inside the fire perimeter after the approximately 187,000 ha Holloway fire in Oregon. Schuyler et al. (2022) identified time lags in responses to sage‐grouse space use after the Holloway fire, and suggested birds may not be fully adapted to the disturbed landscape until 4–5 years postburn. Yet the Holloway fire was a mixed‐severity burn that resulted in a mosaic of intact and burned areas; approximately 75% of the area inside the fire perimeter was still considered habitat for sage‐grouse (Schuyler et al., 2022). By contrast, we observed rapid responses to the Grassy Ridge Fire, which was both smaller in size and extremely high severity, leaving few standing shrubs inside the fire perimeter. Thus, thresholds may exist in the size and severity of disturbance events, beyond which birds may respond faster (e.g., with greater fire severity), or where it may no longer be feasible for sage‐grouse to use remaining habitat outside the burn (e.g., with greater fire size because little habitat remains outside the fire scar). This could account for the different responses observed in our study, whereby sage‐grouse avoided the burned area for nesting immediately after the fire, and instead showed a stronger selection for remaining intact stands of sagebrush to the east and northeast of the fire perimeter.

The structure and composition of remaining vegetation mosaics within burned landscapes may affect breeding habitat selection and survival of nests and broods (Anthony et al., 2021; Brussee et al., 2022). O'Neil et al. (2021) cautioned that rapid habitat change resulting from wildfire, along with site fidelity by nesting females, may limit the ability of sage‐grouse to adapt via modification of space use, contributing to maladaptive habitat selection. O'Neil et al. (2021) further warned that preferential nest‐site selection for pulses of herbaceous vegetation in burned areas could lead to ecological traps, and Anthony et al. (2021) reported higher survival for sage‐grouse nests located in unburned areas relative to those located in remnant vegetation patches within burned areas. O'Neil et al. (2021) allowed for nest selection patterns to vary regionally among populations, yet the inferred ecological traps assumed that habitat selection patterns for a specific resource (e.g., for herbaceous vegetation) do not functionally change after wildfire as the availability of that resource changes across the landscape, whereas recent studies (Smith et al., 2020, this study) suggest this assumption could be tenuous. We observed tradeoff selection during nesting (selection when limited, avoidance when abundant) and increasing use during early brood rearing for perennial herbaceous cover, both of which imply changes in the use of herbaceous vegetation with increased availability. Moreover, we found that selection for sagebrush generally increased in strength when the cover was reduced across the landscape, and sage‐grouse strongly avoided the burned area for nesting. Thus, any potential attraction to the burn due to changes in herbaceous vegetation appeared to be superseded by the lack of shrub cover inside the fire scar.

Irrespective of the potential short‐term responses by individual females, our results demonstrated that functional responses were common in population‐level use and selection of breeding habitat by sage‐grouse. High‐intensity megafire had strong effects on the distribution of available resources, and context‐dependent use and selection of nesting resources were heterogeneous across individual components of habitat. Thus, context‐dependent use of nesting habitat was affected by the overarching influence of large‐scale disturbance on vegetation. Such context specificity in habitat use and selection has strong implications for understanding and predicting how animal populations will use space in a changing environment, and for developing management tools like mapped RSF predictions within and among disturbed landscapes. Our mapped RSFs provided a reasonable fit to observed sage‐grouse space use data and therefore justify the use of these tools for postfire habitat prioritization and restoration in eastern Idaho. Yet our results also imply that extrapolating such predictions into new areas or novel postdisturbance landscapes with differing habitat availabilities could result in unreliable predictions. Nonetheless, increased frequency, severity, and scale of wildfire are consequences of altered disturbance regimes with the capacity to rapidly reshape ecosystems (Abatzoglou & Williams, 2016; Dennison et al., 2014; Westerling et al., 2006). Consequently, our results demonstrate that functional responses can be a useful framework for assessing changes to space use that accompany temporal changes in resource availability, and thus aid large‐scale habitat conservation in a changing world.

## AUTHOR CONTRIBUTIONS

**Bryan S. Stevens:** Conceptualization (equal); data curation (supporting); formal analysis (lead); methodology (lead); writing – original draft (lead); writing – review and editing (equal). **Shane B. Roberts:** Conceptualization (equal); data curation (supporting); funding acquisition (supporting); methodology (supporting); project administration (supporting); writing – review and editing (equal). **Courtney J. Conway:** Conceptualization (equal); funding acquisition (supporting); methodology (supporting); project administration (lead); supervision (equal); writing – review and editing (equal). **Devin K. Englestead:** Conceptualization (equal); data curation (lead); funding acquisition (lead); methodology (supporting); project administration (supporting); writing – review and editing (equal).

## CONFLICT OF INTEREST STATEMENT

The authors declare that they have no competing interests.

## Supporting information


Appendix S1:

## Data Availability

The data analyzed during this study and supporting results tables and figures are archived on Dryad (https://doi.org/10.5061/dryad.x69p8czp0).

## APPENDIX A

Additional tables and figures for describing functional responses in space use for nesting, early brood‐rearing, and late brood‐rearing greater sage‐grouse in eastern Idaho, USA

**TABLE A1:** Final optimally‐predictive resource selection function to predict nest‐site selection by greater sage‐grouse.

Variable	Measurement extent (m)	β
Sagebrush	100	0.11
Sagebrush × Avail	100	−0.44
DI	1000	−0.08
DI^2^	1000	−0.07
DI × Avail	1000	−0.52
Perennial	500	−0.4
Perennial^2^	500	0.07
Perennial × Avail	500	−0.52
Annual	400	−0.15
Litter	1000	0.02
Litter × Avail	1000	−0.08
Bare × Avail	1000	−0.35
Per_grass_cov	‐	−0.09
Per_grass_height	‐	−0.02
Per_grass_height × Avail	‐	−0.31
Forb_height	‐	−0.40
Forb_cov	‐	0.05
Forb_rich	‐	0.27
Forb_rich × Avail	‐	−0.11
CTI	‐	−0.12
Exposure	‐	0.01
Mesic	‐	−0.34
Distance	‐	−0.36
Fire	‐	−1.72

*Note*: Variables included were: sagebrush cover (Sagebrush), Simpson's diversity index of sagebrush cover classes (DI), perennial herbaceous cover (Perennial), annual herbaceous cover (Annual), litter cover (Litter), and bare ground cover (Bare), compound topographic index (CTI), site exposure (Exposure), distance to the closest mesic patch (Mesic), distance to the nearest known lek (Distance), and a binary indicator variable indicating whether a point was inside (1) or outside (0) of the fire perimeter (Fire). The following herbaceous vegetation metrics measured at transects around each nest were also included: perennial grass cover (Per_grass_cov), perennial grass height (Per_grass_height), perennial forb height (Forb_height), forb cover (Forb_cov), and forb richness (Forb_rich). All remotely sensed variables were included at their optimal spatial scale. Moreover, Avail indicates the average available values for a given variable, and thus a variable multiplied by Avail indicates a functional response for that variable was included in the final model. The optimally‐predictive spatial extent for each remotely sensed variable is also provided (Measurement extent). Note also that the LASSO procedure started with 29 coefficients (5 excluded from final model) and used a regularization penalty (λ) of −10.988 (natural log scale).

**TABLE A2:** Final optimally‐predictive resource selection function to predict early brood habitat selection by greater sage‐grouse.

Variable	Measurement extent (m)	β	βs
Sagebrush	1000	0.36	0.28
Sagebrush^2^	1000	−0.90	−0.88
Sagebrush × Avail	1000	0.68	0.73
Sagebrush^2^ × Avail	1000	−0.07	−0.02
DI	800	−0.04	−0.004
DI^2^	800	−0.62	−0.61
DI × Avail	800	0.74	0.68
DI^2^ × Avail	800	−0.001	−0.03
Perennial	200	0.16	0.14
Perennial × Avail	200	0.04	−0.004
Annual	500	−0.24	−0.26
Annual × Avail	500	0.07	0.08
Litter	300	−0.0004	0.00005
Litter^2^	300	−0.07	−0.05
Bare	100	0.65	0.63
Bare^2^	100	−0.76	−0.77
Bare × Avail	100	0.67	0.67
Rough	‐	−0.05	−0.09
CTI	‐	−0.07	−0.08
Exposure	‐	0.09	0.04
Mesic	‐	−0.01	−0.02
Fire	‐	−0.03	−0.07

*Note*: Variables included were: sagebrush cover (Sagebrush), Simpson's diversity index of sagebrush cover (DI), perennial herbaceous cover (Perennial), annual herbaceous cover (Annual), litter cover (Litter), and bare ground cover (Bare), topographic roughness (Rough), compound topographic index (CTI), site exposure (Exposure), distance to the closest mesic grass patch (Mesic), and distance outside of the fire perimeter (Fire). All remotely sensed cover variables were included at their optimal spatial scale (Measurement extent). Moreover, Avail indicates the average available values for a given variable, and thus a variable multiplied by Avail indicates a functional response for that variable was included in the final model. Also shown are coefficient estimates from the sensitivity analyses that reran the analysis using no more than 3 (randomly selected) use points per day for each individual bird (β_s_). Note also that all coefficients included in the LASSO procedure were retained, and the optimal regularization penalty (λ) was −15.680 (natural log scale).

**TABLE A3:** Final optimally‐predictive resource selection function to predict late brood habitat selection by greater sage‐grouse.

Variable	Measurement extent (m)	β	β_s_
Sagebrush	100	0.23	0.23
Sagebrush^2^	100	−0.42	−0.45
Sagebrush × Avail	100	0.47	0.47
Sagebrush^2^ × Avail	100	−0.30	−0.31
DI	700	0.06	0.08
DI^2^	700	−0.44	−0.41
DI × Avail	700	0.40	0.37
DI^2^ × Avail	700	−0.35	−0.36
Perennial	100	0.03	0.03
Perennial^2^	100	0.07	0.07
Perennial × Avail	100	0.003	−0.005
Annual	500	−0.34	−0.34
Annual^2^	500	0.06	0.06
Annual × Avail	500	0.03	0.03
Litter	300	0.07	0.09
Litter × Avail	300	−0.02	−0.007
Bare	300	−0.72	−0.72
Bare^2^	300	−0.57	−0.55
Bare × Avail	300	−0.46	−0.39
Bare^2^ × Avail	300	0.59	0.55
Rough	‐	−0.29	−0.31
CTI	‐	−0.003	−0.005
Exposure	‐	−0.21	−0.22
Mesic	‐	−0.06	−0.06
Fire	‐	0.12	0.11

*Note*: Variables included were: sagebrush cover measured at 100 m (Sagebrush), Simpson's diversity index of sagebrush cover classes measured at 700 m (DI), perennial herbaceous cover measured at 100 m (Perennial), annual herbaceous cover measured at 500 m (Annual), litter cover measured at 300 m (Litter), and bare ground cover measured at 300 m (Bare), topographic roughness (Rough), compound topographic index (CTI), site exposure (Exposure), distance to the closest mesic grass patch (Mesic), and distance outside of the fire perimeter (Fire). All remotely sensed cover variables were included at their optimal spatial scale (Measurement extent). Moreover, Avail indicates the average available values for a given variable, and thus a variable multiplied by Avail indicates a functional response for that variable was included in the final model. Also shown are coefficient estimates from the sensitivity analyses that reran the analysis using no more than 3 (randomly selected) use points per day for each individual bird (β_s_). Note also that all coefficients included in the LASSO procedure were retained, and the optimal regularization penalty (λ) was −15.531 (natural log scale).
