# Supplementary material for: Effects of large‐scale disturbance on animal space use: Functional responses by greater sage‐grouse after megafire
Source: Ecol Evol. 2023 Apr 7;13(4):e9933. doi: 10.1002/ece3.9933 (PMC10082181; doi:10.1002/ece3.9933)
Supplement: Supplementary file 1 — Appendix S1: [file ECE3-13-e9933-s001.docx]

**Supplemental description of methods to generate a composite RSF to map relative probability of selection by female sage-grouse over the breeding season.**

We developed a composite map of relative probability of selection by greater sage-grouse during the breeding season, pre and post fire. This predictive map was developed for conservation and management decision support, to understand the implications of fire for space use and to aide in post fire conservation planning. The approach we used to integrate predictions across models (i.e., nest selection, early and late brood selection RSFs) was motivated by and conceptually similar to the scale-integrated RSF mapping approach described by DeCesare et al. (2012), but differed in several important ways. Here we highlight the DeCesare et al. (2012) method and describe our approach merging predictions into a single map, relative to their work. DeCesare et al. (2012) described the scale-integrated relative probability of selection ($w_{integrated}$) as the joint probability of selection for a given map pixel (as a function of resources contained therein) based on RSFs developed at 3 spatially nested scales (scales 1-3, going from broader to finer spatial extents):

$w_{integrated}=P\left( S_{1},S_{2},S_{3} \right)=P(S_{1})\times P(S_{2}|S_{1})\times P(S_{3}|S_{2})$,

where availability was sampled for each nested scale conditional on the binary outcome of the scale above it. Specifically, availability was sampled such that RSFs for scales 2 and 3 predicted $P(S_{2}|S_{1}=1)$ and $P(S_{3}|S_{2}=1)$, respectively, by only drawing random available samples from spatial regions where those conditions were met. That is, selection for a pixel at scale 1 had already occurred for the RSF that predicted $P(S_{2})$, and selection for a pixel at scale 2 had already occurred for the RSF that predicted $P(S_{3})$. Given the way that availability was sampled, conditional on outcomes at spatially nested scales, the scale-integrated predictions reduced to:

$$w_{integrated}=P\left( S_{1},S_{2},S_{3} \right)=P\left( S_{1} \right)\times P\left( S_{2} \right)\times P\left( S_{3} \right).$$

Thus $w_{integrated}$ represented the relative probability that a pixel was used as a function of predictions from RSFs developed at 3 nested spatial extents (i.e., probability that a pixel is used at scale 1, scale 2, and scale 3, respectively). Note however that the binary outcome assumed for availability sampling was not actually conditioned upon when DeCesare et al. (2012) generated mapped predictions. Instead, predictions from each model were continuous in nature and did not condition on any binary outcomes observed at larger scales. Doing so would have required a binary prediction threshold to be developed for the 2 largest scales, in order to convert continuous predictions into a binary outcomes that lower-scale predictions could condition upon (i.e., lower scale predictions would be zero if higher scale outcome was 0, and continuous but positive otherwise). Therefore $w_{integrated}$ was approximated by multiplying the relative probability (*w*) predictions generated independently for each RSF, one developed separately for each of the 3 scales.

For our sage-grouse study, we generated an integrated map of RSF predictions that were integrated across the process-specific RSFs developed for nest site selection, and early and late brood rearing selection. However, these models were not nested spatially like those od DeCesare et al. (2012). Specifically, our models predicted the relative probabilities $P(S_{nest})$, $P(S_{early}|S_{nest})$, and $P(S_{late}|S_{nest})$, respectively. Availability sampling for nest sites occurred at the study area level (i.e., for the entire breeding population), whereas availability (and use) for brood rearing were sampled within buffers (1.7 km and 8 km) that conditioned, for each individual animal, on the nest location (i.e., buffers around nest sites). Because availability sampling for brood selection conditioned on the nest site, and also because both the early and late brood RSFs contained interaction terms where strength of selection for resources changed based on locally available conditions around each nest (i.e., the functional responses, $\beta\left( x_{i}\times\bar{A}_{x} \right)$, where $x_{i}$ is the value of covariate *x* at use location *i* and $\bar{A}_{x}$ is the average value of *x* across available locations [within 1.7 or 8 km] around each individual’s nest), this means that pixel-scale RSF predictions from brood selection models implicitly condition on a pixel being used also as a nest site. That is, when mapping predictions across the study area, available conditions surrounding each pixel were generated using moving window analyses that averaged covariates within 1.7 and 8 km, respectively (otherwise predictions could not take into account locally-varying availability of resources and subsequent functional response interactions). Given the way that availability data sampling conditioned on the nest location, this approach for quantifying locally-varying availability implicitly assumes that a pixel was also a nest site. Therefore stand-alone predictions mapped individually onto the study area from the early and late brood models implicitly assumed that all pixels have an equal probability of use as a nest location (and hence broods are equally likely to occur anywhere on the landscape), which is not accurate. Also note that $P(S_{early}|S_{nest})$ and $P(S_{late}|S_{nest})$ were conditionally independent (i.e., after accounting for nest location availability sampling they were not linked explicitly). Consequently, for our mapped predictions the product of all 3 RSFs approximated

$w_{composite}=P\left( S_{nest},S_{early},S_{late} \right)=P(S_{nest})\times P(S_{early}|S_{nest})\times P(S_{late}|S_{nest})$,

where $w_{nest}\times w_{early}\times w_{late}$ was used to approximate $P\left( S_{nest},S_{early},S_{late} \right)$, the relative probability of selection for a pixel across all periods of the breeding season. In effect, the composite predictions weight the probability of use during the brood period by the probability that a pixel was used for nesting (and hence the brood is more likely to be in the area to begin with), which is conceptually similar to using broad-scale population-level RSFs to weight local-scale mapped RSF predictions (e.g., DeCesare et al. 2012), and also substantially improved fit of the mapped predictions to the observed use data from this study (see Results section). As such, the predictions from our composite map represent predicted intensity of use over the breeding season, where brood use is weighted by probability of use for by nesting females, which is distinct from, but conceptually motivated by, the scale-integrated RSF predictions presented originally by DeCesare et al. (2012).

Literature Cited

DeCesare, N.J., M. Hebblewhite, F. Schmiegelow, D. Hervieux, G.J. McDermid, L. Neufeld, M. Bradley, J. Whittington, K.G. Smith, L.E. Morgantini, M. Wheatley, and M. Musiani. 2012. Transcending scale dependence in identifying habitat with resource selection functions. Ecological Applications 22:108–1083.
